# Global pattern of trends in incidence, mortality, and mortality-to-incidence ratio rates related to liver cancer, 1990–2019: a longitudinal analysis based on the global burden of disease study

**DOI:** 10.1186/s12889-022-12867-w

**Published:** 2022-03-29

**Authors:** Maedeh Amini, Mehdi Azizmohammad Looha, Elaheh Zarean, Mohamad Amin Pourhoseingholi

**Affiliations:** 1 Basic and Molecular Epidemiology of Gastrointestinal Disorders Research Center, Research Institute for Gastroenterology and Liver Diseases, Shahid Beheshti University of Medical Sciences, Tehran, Iran; 2 Precision Medicine, School of Clinical Sciences at Monash Health, Monash University, Clayton, VIC Australia; 3 Gastroenterology and Liver Diseases Research Center, Research Institute for Gastroenterology and Liver Diseases, Shahid Beheshti University of Medical Sciences, Tehran, Iran

**Keywords:** Liver cancer, Incidence, Mortality, Mortality-to-incidence ratio, Human development index, Trend, Multilevel analysis

## Abstract

**Background:**

Liver cancer (LC) is considered as one of the most dominant malignant tumors which ranked 4^th^ and 6^th^ in terms of global mortality and incidence, respectively. This work aimed to investigate the global temporal trends in LC mortality-to-incidence ratio (MIR) and its components, with a particular focus on examining long-term effect of human development index (HDI) on these metrics in a 30-year follow-up.

**Methods:**

The age-standardized LC incidence and mortality data were derived from the global burden of disease (GBD) study 2019. We first leveraged joinpoint piecewise linear regression analysis to ascertain time trends in LC incidence, mortality, and MIR complement [1-MIR] and the average annual percentage change (AAPC) of the rates over the period 1990–2019. Then, the association between the metrics and HDI was explored through longitudinal multilevel models (LMMs).

**Results:**

The incidence rates paralleled the mortality rates worldwide and they had similar significant monotonic decrementing trends with AAPC values of − 1.10% (95% confidence interval (CI): − 1.40, − 0.90%) and − 1.40% (− 1.50, − 1.30%), respectively from 1990 to 2019. The [1-MIR] rates were around 0 and showed an increasing pattern from 1.70 to 8.10 per 100,000 people (AAPC, 4.90%) at the same period of time. Results from the LMMs displayed that the majority of the variation lies at the country level accounted for about 88% of the total variance. Moreover, our analysis supported that the HDI was negatively associated with either incidence or mortality over time (*p* < 0.05).

**Conclusions:**

Our findings highlighted that the global long-term temporal trends of LC incidence and mortality decreased slightly during 1990–2019 which may reflect improved therapeutic strategies and public health interventions. Besides, the low rates of [1-MIR] revealed the five-year relative survival rate was poor implying LC is diagnosed late in its development. Thereby, the policymakers’ focus must be on early screening and detection of liver cancer.

## Background

Cancer is recognized as a serious and prevalent public health problem and its incidence and mortality rates are rapidly rising continuously around the world [1]. Liver cancer (LC) is still one of the most dominant malignant tumors in many countries, ranking 4^th^ and 6^th^ among all cancers in terms of overall mortality and incidence, respectively [2]. The disease imposed a large burden on a number of countries because of high prevalent and mortality. Estimates from the GLOBOCAN 2020 revealed that over 830,000 inhabitants died from LC, making it the second leading cause of cancer death in males and sixth in females across the globe. On the other hand, the LC incidence has been indicated to be growing in most regions of the world such that an estimated 905,677 incident cases was reported in 2020, ranking 5th in men and ninth in women. Generally, both the incidence and mortality rates among males are two to three times greater than those among females in many areas of the world [1]. Of note, the prognosis for liver cancer is among the poorest of all cancers and it is usually diagnosed in advanced stages. Only a 5–30% survival rate due to LC in the world during 2000–2014 despite rapid development of diagnostic and therapeutic techniques [2].

To estimate general cancer survival, accurate population-based cancer registries are required. However, lack of active monitor cancer epidemiology, particularly in low- and medium-resource countries may fail the calculation of survival parameters [3, 4]. Previous literature has therefore proposed a novel measure of the completeness of a cancer registry when considering the quality of cancer care and reporting. It was originally referred to “deaths in period”, but later became known as the mortality-to-incidence ratio (MIR) which its value can serve as a proxy for 1-survival in various populations. This index may have role in expanding interpretation of the relationship between the two epidemiological measurements as well as clarify the difference between the results of cancer management and health care systems. Recently, it was found a strong association between five-year relative survival rate and the MIR complement [1-MIR]. Moreover, the indicator has been used to describe cancer disparities and also ascertain whether a country has a higher mortality than might be expected according to its incidence [5]. Nonetheless, temporal patterns of [1-MIR] from LC in different countries have been not undertaken to explicate its potential usefulness as a surveillance tool for LC. While, an understanding of secular trends of [1-MIR] is a clue to evaluating the effect of present interventions and guiding future policy.

As with earlier reports, the LC incidence rates have increased substantially sharply in some low-risk areas such as Western Europe, Australasia, and North America in recent decades [6, 7]. A previous global study showed that the incidence of LC was 5.1 and 6.6 per 100,000 persons in Europe and North America, respectively in 2018, whilst the rates were 11.4 and 8.4 in Asia and Africa, respectively, suggesting the high incidence in less developed regions [8]. Liver cancer mortality trends followed similar pattern to incidence in regions of the world which have experienced relatively low rates, e.g. the United States of America, Canada, United Kingdom, and most of Central and Northern European countries over the same period [6, 9]. Additionally, some evidence declared that there are remarkable variations in LC incidence and mortality trends in various countries [10, 11]. Socioeconomic factors (e.g. life expectancy and access to health care) may have contributed to these differences in trends among countries [12]. One of the most important known indicators for level of social and economic progress is human development index (HDI) which is a composite index focusing on three basic dimensions of human development. It combines a gross national income (GNI) per capita as determined by the World Bank, mean life expectancy of the population at birth, and mean or expected years of schooling of that citizens of country [13]. Numerous published studies supported that the cancer disparities might be associated with levels of HDI [14, 15].

Most previous epidemiological papers on liver cancer have evidenced that the secular trends in incidence and mortality are highly heterogeneous across the world. Modeling this unobserved heterogeneity which explain subject-level effects is one way to accommodate the correlation of the repeated responses over time [16]. Statistical investigators developed a wide variety of techniques to detect and analyze individual heterogeneities and heterogeneities between the groups. Among these, hierarchical or multilevel models, as an extension of traditional random effects models, have been popular to provide a natural manner to decompose complex patterns of variability related to hierarchical or nested structures [17]. A noticeable feature of this methodology is that inference can be made on the variability at each level in the data hierarchy. Statisticians declared that failure to take account of such structures in traditional models may cause incorrect inferences [18].

From the 1990s to today, although few studies from a variety of international sources assessed long-term trends in burden of disease attributable to LC via advanced statistical methods, their breakdown was mainly limited to specific regions and populations. In this report, we filled these voids utilizing data from the Global Burden of Disease (GBD) study 2019 collected annually to allow patterns in trends over a 30-year time period to be observed [19]. Accordingly, no evidence yet supported longitudinal association between the HDI levels and the mentioned burden indices of patients with LC. Better understanding of this complex relationship may help providers to prioritize management in the context of cancer care and serve as a guide for the improvement function and quality of life. As a whole, our primary goal was to elucidate and analyze time patterns in [1-MIR] rates and its components based on gender, super-region, age groups, and total populations whether there have been any changes in the patterns during the years from 1990 to 2019. Our secondary purpose was to explore the influence of HDI on liver cancer outcomes over the specified time period and to determine the variations at the different level of hierarchical structure of GBD data applying multilevel modelling approach.

## Methods

### Study data

In brief, the GBD 2019 data for the disease burden of liver cancer were derived from the Global Health Data Exchange (GHDx) query tool. The GBD study 2019 was developed and coordinated by the Institute for Health Metrics and Evaluation (IHME) at the University of Washington, which provides rigorous and comparable measurements of important health problems around the world. The GBD 2019 systematically and comprehensively estimated 286 causes of death, 369 diseases and injuries, and 87 risk factors for 204 countries and territories from a variety of relevant data sources, including household surveys, censuses, vital statistics, and civil registrations [19]. The cancer burden information in the GBD study was estimated based on multiple national cancer registry systems and aggregate database of cancer registries, such as Cancer Incidence in Five Continents, Surveillance, Epidemiology, End Results, and NORDCAN. Detailed information on original data sources used in the current study can be retrieved on the GBD 2019 Data Input Sources Tool website (http://ghdx.healthdata.org/gbd-2019/data-input-sources).

Herein, the variables obtained from GBD statistics contained annual liver cancer incidence and mortality rates in groups stratified by different genders, regions, countries, ages (0–14 years, 15–49 years, 50–69 years, and = > 70 years), as well as the corresponding age-standardized rates. Incidence rate per 100,000 people was defined as the number of new cases divided by the population size. Mortality rate per 100,000 person-years was defined as the number of annual deaths divided by the entire population size. Meanwhile, the MIR complement was computed from subtracting from the result obtained by dividing the crude rate of LC mortality for a given year by the corresponding crude rate of incidence during the same time period in a specific population multiplied by 100. This ratio is ranged between 0 and 100%, where 0% indicates an extremely poor survival and 100% an excellent survival [4].

Geographically, in the GBD framework, the world was separated into seven super-regions and 21 regions. In GBD study, the countries and territories were categorized as low, low-medium, medium, high-medium, and high regions according to the classification of socio-demographic index (SDI). Likewise, according to HDI as the gold standard for international comparisons of development, countries are assigned into four categories (low, medium, high, and very high). The united nation development programme has defined the HDI to measure the mean achievement in a country in three basic indices as long and healthy life, access to knowledge and awareness, and decent standard of living and promotion in life expectancy [20]. The HDI for each country can be obtained from the United Nations Development Program, Human Development Report Office. It is important to note that the countries with HDI values of less than 0.788 were defined as “less-developed” and those with HDI values of 0.788 or higher as “more developed” [21]. We confirm that all methods were performed in accordance with the relevant guidelines and regulations.

In the current study, the considered outcomes were the LC incidence, mortality, and [1-MIR] in the period 1990–2019. Accordingly, the development index was utilized as a binary independent predictor variable taking the value of 0 = less-developed or the value of 1 = more developed for the metrics in the statistical modeling process.

#### Statistical analysis

At first, we provided the information about the LC incidence, mortality, and [1-MIR] rates and 95% uncertainty intervals (UIs) expressed per 100,000 persons mainly to describe the period-pattern in different super-regions, genders, and age groups every 6 years. The 95% UIs were calculated as taken the 2.5^th^ and 97.5^th^ percentiles values and all estimates were reported next to each point estimate. The analyses for this literature contained of three steps, described in detail below.

### Step 1: latent growth curve modeling

The trends of [1-MIR] rates and its components in 30 years from 1990 to 2019 were evaluated via latent growth curve models (LGCMs). This is a powerful analytic method within the structural equation modeling framework to determine longitudinal change over time as an underlying latent process. In analyzing this process, change is modeled as a function of time and is denoted via the specification of latent (i.e., unobserved) variables referred to as growth factors. Latent intercept and slope as the growth factors are estimated according to the individual trajectories. Growth factors can provide an estimate of the average trajectory and individual variations around the trajectory during the study period. This rate of change with an intercept become the variables of interest in LGCM modeling [22]. In general, the corresponding LGCM is specifies as following equations

1.1 $${y}_{ti}={\lambda}_{0t}{\eta}_{0i}+{\eta}_{1i}{\lambda}_{1t}+{\gamma}_{2t}{x}_{ti}+{\varepsilon}_{ti},$$

1.2 $${\eta}_{0i}={\eta}_0+{\gamma}_0{z}_i+{\varsigma}_{0i},$$

1.3 $${\eta}_{1i}={\eta}_1+{\gamma}_1{z}_i+{\varsigma}_{1i},$$

in which Eq. (1.1) is the within subject model such that *y*_*ti*_ is the observation (the considered disease outcome) for *i* ^th^ country (*i* = 1, 2, …, 184) at time point t (*t* = 1, 2, …, 30), *η*_0*i*_ and *η*_1*i*_ are latent growth factors. *λ*_0*t*_ denotes a constant equal to the value of 1 and *λ*_1*t*_ is the time of measurement. *ε*_*ti*_ is a composite error term at time *t*. Eqs. (1.2) and (1.3) are the between subject models, in which *η*_0*i*_ and *η*_1*i*_ are the two random coefficients. Moreover, *η*_0_ and *η*_1_ represent the model estimated overall mean level of the initial outcome and the average rate of outcome change over the entire analysis period, respectively. *ς*_0*i*_ and *ς*_1*i*_ are error terms indicating between individual variations regarding the outcome growth trajectory. *γ*_2*t*_ is the effect of the time-varying covariate *x*_*ti*_. *γ*_0_ and *γ*_1_ represent the effects of the time-invariant covariate on the initial level and linear slope [23]. The lavaan package of the open-source R 4.1.1 software was performed to conduct LGCM analysis [24].

### Step 2: Joinpoint regression

It is often would be valuable for clinical researchers to aware of change points in a trend and the evaluation of whether there are significant changes in the observed trend at particular points (joinpoints). Here, we employed joinpoint regression analysis using the joinpoint statistical software version 4.8.0.1 to estimate annual percent changes (APCs) and their averages (AAPCs) in [1-MIR] and its components over the study period, which are readily interpretable as well as directly comparable across different strata [25, 26]. The software calculates the trends, starting with the minimum joinpoint of 0 (representing a straight line), followed by test (Monte Carlo permutation) for the statistical significance of the changes after adding more joinpoints along with their 95% confidence intervals (CIs). The APC was computed as APC_*i*_ = [(exp(*β*_*i*_)-1)] × 100 where *β*_*i*_ states the slope of the trend segment. Subsequently, the AAPC was calculated as a geometrically weighted average of the different APCs with weights being equivalent to the length of each segment during the specified time interval [27].

### Step 3: longitudinal multilevel modeling

Hierarchically structured data are frequently encountered in various fields of scientific investigation. Over the past 20 years, multilevel analysis as an extension of the generalized linear mixed models (GLMMs) considers the existence of nested data structures, wherein certain variables specify variation between distinct units which represent groups. In this model, the hierarchical structure is referred to the level. The nesting levels are usually numbered from the lowest to highest level so that level 1 units nested within level 2 units nested within level 3 units, etc. that longitudinal data are a special case of multilevel data and arise when individuals are measured several times during an observation period [28]. One of the important benefits of longitudinal data analysis is to determine the diversity between units both within the level of the response and in changes between different times. Longitudinal multilevel modelling have become popular in the analysis of within subject and between subject changes by discriminating two questions: how individuals change over time and how these changes vary across individuals. The resulting longitudinal data in the simplest form would be nested at two level, with repeated measures over time (level 1 or the occasion level) treated as nested within participants (level 2 or the individual level [29]. Because the data used in this literature had a hierarchy structure, longitudinal multilevel modelling approach was implemented to investigate the connection between HDI (development status) and incidence, mortality, and [1-MIR] over time. The longitudinal multilevel and LGC approaches can be utilized to formulate equivalent models, but there is some differences between them. A longitudinal four-level model with random intercept for the considered outcome is given by

2 $${Y}_{ij k z}={\beta}_{0 jk\omega}+{\beta}_1{t}_{ij k\omega}+{\beta}_2{x}_{ij}+{\beta}_3\left({x}_{ij}\times {t}_{ij k\omega}\right)+{b}_{\omega}^{(4)}+{b}_{k\omega}^{(3)}+{b}_{jk\omega}^{(2)}+{\varepsilon}_{ij k\omega},$$

where *Y*_*ijkω*_ indicate the repeated response variable of the *i* ^th^ (*i* = 1, 2, …, 30) level 1 unit within the *j* ^th^ (*j* = 1, 2, ..., 184) level 2 cluster within the *k* ^th^ (*k* = 1, 2, …, 21) level 3 cluster within the *ω* ^th^ (*ω* = 1, 2, …, 7) level 4 cluster; *t*_*ijkω*_ denotes the time since baseline for the *i* ^*t*h^ observation on the *j* ^th^ country in the *k* ^th^ region nested within *ω* ^th^ super-region. *x*_*ij*_ is the explanatory variable (i.e. HDI) for the *i* ^th^ observation in the *j* ^th^ country. *β*_0_ is the initial mean of disease outcome rate and *β* = (*β*_1_, *β*_2_, *β*_3_) reflects the regression coefficient and describe the effect of covariates on the mean response. The term *x*_*ij*_ × *t*_*ijk*_ is the cross-level interaction effect. *ε*_*ijkω*_ denote the error terms; ${b}_{\omega}^{(4)}$ is a random super-region effect, ${b}_{k\omega}^{(3)}$ is a random region effect and ${b}_{jk\omega}^{(2)}$ is a random effect country effect. As a side note, it is assumed that *Y*~*N*(*μ*, *σ* ^2^), ${b}_{\omega}\sim N\left(0,{\sigma}_{\omega}^2\right),{b}_{k\omega}\sim N\left(0,{\sigma}_{\upsilon}^2\right)$, ${b}_{jk\omega}\sim N\left(0,{\sigma}_u^2\right)$, and ${\varepsilon}_{ijk\omega}\sim N\left(0,{\sigma}_e^2\right)$. Additionally, we assumed that the random effects were independent of each other. This is the simplest type of multilevel model wherein the intercept is allowed to differ between clusters whereas the coefficient *β* across clusters would be the same [30].

To examine the amount of dependency among the country observations within the super-regions, the intraclass correlation coefficient (ICC) was utilized. The ICC is interpreted as a measure of the proportion of variance of a given response variable explained by a factor of interest in an analysis of variance model where it measures the relative homogeneity within groups whose value generally lies between 0 and 1. Larger values of the ICC are suggestive of a superior impact of clustering and observations in the same cluster are more closely related. As the ICC increases in value, using multilevel modelling strategies in data analysis should be taken to account. In a four-level model, the index at the country, region, and super-region levels can be estimated by the following proposition

3.1 $${\mathrm{ICC}}_{\mathrm{country}}=\frac{\sigma_u^2}{\sigma_{\omega}^2+{\sigma}_{\upsilon}^2+{\sigma}_u^2+{\sigma}_e^2}$$

3.2 $${\mathrm{ICC}}_{\mathrm{region}}=\frac{\sigma_{\upsilon}^2}{\sigma_{\omega}^2+{\sigma}_{\upsilon}^2+{\sigma}_u^2+{\sigma}_e^2}$$

3.3 $${\mathrm{ICC}}_{\mathrm{super}-\mathrm{region}}=\frac{\sigma_{\omega}^2}{\sigma_{\omega}^2+{\sigma}_{\upsilon}^2+{\sigma}_u^2+{\sigma}_e^2}$$

where ${\sigma}_{\upsilon}^2$ is the component of variation because of variability among super-regions, ${\sigma}_u^2$ is the component of variation because of variability among countries nested within super-regions, and ${\sigma}_e^2$ is the residual component of variation owing to variability among lower-level units. If the ICC equals 0, it suggests that all the observations are independent of one another. By contrast, if the ICC equals 1, it implies that all the responses from observations in all clusters are exactly the same and there is high correlation among them [18]. According to a study, an ICC of ≥2.0% shows the need to do the multilevel model for analysis of data [31]. All models for the burden of LC applied the following hierarchy: repeated measures of incidence, mortality, and [1-MIR] (level 1 units) as nested within 184 countries (level 2 units), countries nested within 21 regions (level 3 units), and regions nested within 7 super-regions (level 4 units). By fitting 4-level models, the effect of development status on the desired outcomes was investigated. The multilevel analysis based on restricted maximum likelihood estimation (REML) approach was performed using R version 4.1.1 (lme4 package) to estimate the parameters [32]. A *P* value less than 0.05 was regarded as statistically significant.

## Results

### Temporal trends and latent growth curve analysis of LC incidence, mortality, and [1-MIR]

**Fig. 1:**
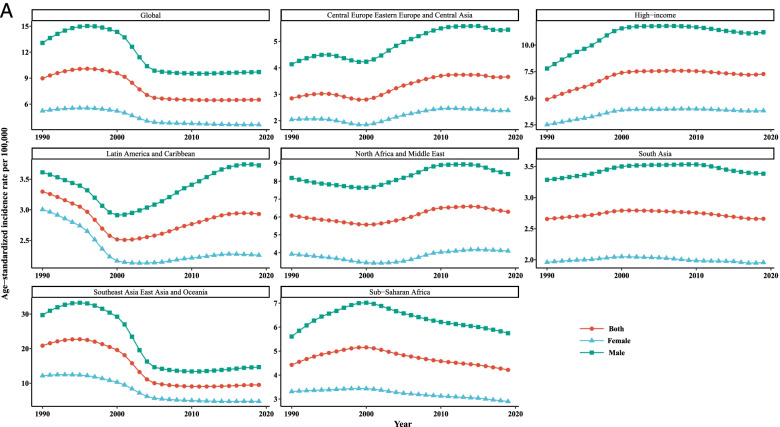

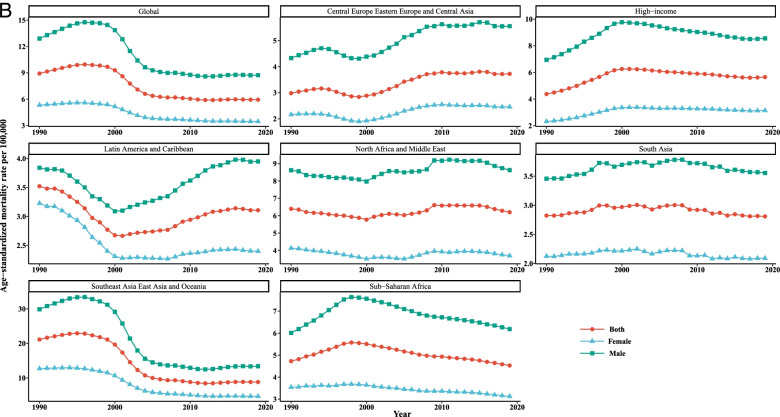

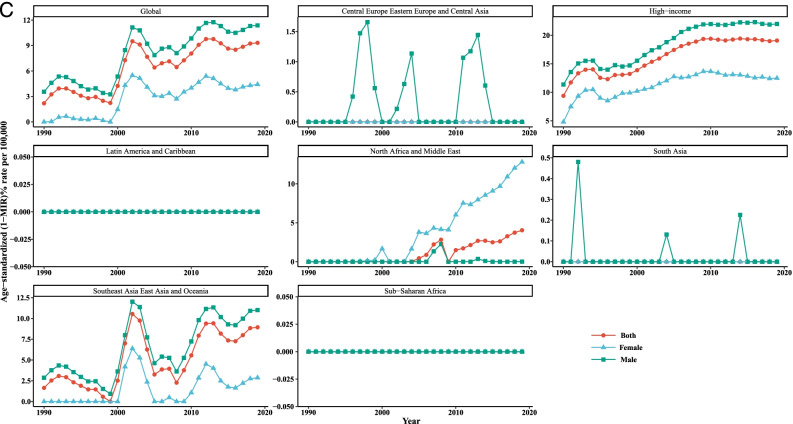
Mean age-standardized temporal trends in liver cancer (**A**) incidence, (**B**) mortality, and (**C**) [1-MIR] per 100,000 by gender in each super-region, 1990–2019

The LC age standardized incidence, mortality, and [1-MIR] rates per 100,000 people along with LGCM parameters demarcated by gender from 1990 to 2019 (in 6-year intervals) in various super-regions and the whole world are reported in Table 1 and the growth trajectories are depicted in Fig. 1. In total, the incidence rates for all super-regions showed that males experienced higher rates of incidence and mortality compared with females during time intervals in the last 30 years. For females, the age-standardized incidence rate (ASIR) was the highest in Southeast Asia, East Asia, and Oceania (SAEAO), with 12.18 per 100,000 in 1996 and lowest in South Asia (SA), with 1.95 per 100,000 in 2019. For males, the highest ASIR was observed in SAEAO (33.06/100,000) in 1996 and lowest in Latin America and Caribbean (LAC) (2.95/100,000) in 2002. Notably, based on LGCM results, statistically significant intercepts for all super-regions have been identified, which implied the mean incidence, mortality, and [1-MIR] rates were substantially far from zero in 1990. With an accurate look at the mean intercept estimates for males, this is observable that SAEAO and LAC had the highest and lowest ASIR of LC with 12.63 and 3.40 per 100,000 people, respectively at the starting year of the study. Similarly, by comparing the intercepts for females in 1990 one can conclude that the highest and lowest overall mean levels of initial LC ASIR were related to SAEAO and LAC countries with 6.11/100,000 and 2.11/100,000, respectively. On the other hand, a positive or negative slope reveals the rising or falling trend in these super-regions over the period 1990–2019. For males and females, downward significant trends were visible in SAEAO, Sub-Saharan Africa (SSA), and High Income (HI), while LAC indicated upward trends. In other super-regions, the average rates of change (slope) in LC ASIR has not been statistically significant for both genders over time (*p* > 0.05). Intuitively, from Fig. 1, we observed that the ASIRs followed a similar pattern of trends for men and women in each super-region during the entire study period. Moreover, among 7 super-regions showing different trends in liver cancer-related ASIR, LAC and SAEAO were the areas that had the most rapid decrement until 2000 and 2006, respectively for each sex. In males, the ASIRs displayed a greatest growing trend in LAC (from 2002 till 2016), Central Europe, Eastern Europe, and Central Asia (CEEECA) (from 2001 to 2011), SSA (from 1990 to 1999), and HI (from 1990 till 2000). However, the LC ASIR has remained rather stable in SA for both men and women during the whole time period from 1990 to 2019.

**Table 1:** Age standardized incidence, mortality, and 1-MIR rates per 100,000 individuals as well as LGCM results stratified by gender and super- region, 1990–2019

Super- region	Metric	Gender	Year						LGCM estimates`	
			1990	1996	2002	2008	2014	2019	Intercept	Slope
	Incidence									
		Female	2.03(1.95,2.11)	1.99(1.92,2.07)	1.96(1.88,2.03)	2.37(2.27,2.47)	2.43(2.33,2.53)	2.38(2.16,2.64)	3.58^*^	0.08^**^
		Male	4.13(3.99,4.6)	4.44(4.29,4.58)	4.46(4.34,4.57)	5.31(5.15,5.45)	5.58(5.39,5.77)	5.44(4.79,6.07)	8.31^*^	0.11^**^
		Both	2.84(2.74,2.92)	2.97(2.87,3.06)	2.96(2.88,3.04)	3.57(3.45,3.68)	3.73(3.60,3.85)	3.65(3.33,3.98)	5.62^*^	0.09^**^
CEEECA	Mortality									
		Female	2.15(2.06,2.24)	2.07(1.99,2.13)	2.02(1.94,2.10)	2.49(2.38,2.59)	2.51(2.38,2.60)	2.45(2.21,2.69)	3.68^***^	0.11^*^
		Male	4.33(4.17,4.46)	4.55(4.42,4.68)	4.56(4.43,4.68)	5.53(5.33,5.71)	5.62(5.42,5.80)	5.55(4.90,6.24)	8.62^*^	0.15^*^
		Both	2.98(2.86,3.06)	3.03(2.94,3.11)	3.02(2.93,3.10)	3.71(3.57,3.82)	3.77(3.63,3.87)	3.72(3.39,4.07)	5.76^*^	0.12^*^
	1-MIR									
		Female	0.00(0.00,0.00)	0.00(0.00,0.00)	0.00(0.00,0.00)	0.00(0.00,0.00)	0.00(0.00,0.00)	0.00(0.00,0.00)	–	–
		Male	0.00(0.00,0.00)	0.004(0,0.008)	0.002(0,0.003)	0.00(0.00,0.00)	0.006(0.005,0.007)	0.00(0.00,0.00)	0.005^*^	0.001^**^
		Both	0.00(0.00,0.00)	0.00(0.00,0.00)	0.00(0.00,0.00)	0.00(0.00,0.00)	0.00(0.00,0.00)	0.00(0.00,0.00)	–	–
	Incidence									
		Female	2.50(2.37,2.59)	3.25(3.04,3.38)	3.94(3.64,4.10)	3.99(3.62,4.18)	3.87(3.47,4.09)	3.82(3.35,4.25)	2.25^***^	−0.08^***^
		Male	7.79(7.59,7.95)	9.96(9.67,10.20)	11.74(11.39,11.97)	11.76(11.29,12.09)	11.32(10.78,11.66)	11.20(10.05,12.50)	5.63^***^	−0.15^***^
		Both	4.88(4.74,4.99)	6.29(6.05,6.45)	7.51(7.20,7.68)	7.58(7.17,7.81)	7.33(6.90,7.58)	7.27(6.55,8.01)	3.78^***^	−0.10^***^
HI	Mortality									
		Female	2.32(2.18,2.40)	2.88(2.68,2.98)	3.38(3.10,3.52)	3.30(2.96,3.48)	3.19(2.82,3.37)	3.14(2.78,3.35)	2.21^***^	−0.05^***^
		Male	6.94(6.76,7.10)	8.60(8.33,8.78)	9.68(9.36,9.90)	9.17(8.76,9.46)	8.68(8.25,8.95)	8.55(7.94,8.97)	5.37^***^	−0.09^***^
		Both	4.38(4.23,4.48)	5.44(5.20,5.56)	6.24(5.94,6.40)	5.98(5.61,6.18)	5.71(5.31,5.91)	5.65(5.23,5.95)	3.63^***^	−0.08^***^
	1-MIR									
		Female	0.04(0.03,0.06)	0.09(0.08,0.10)	0.10(0.12,0.11)	0.13(0.11,0.14)	0.13(0.14,0.15)	0.12(0.11,0.16)	0.12^***^	0.002^***^
		Male	0.11(0.10,0.12)	0.13(0.11,0.14)	0.17(0.16,0.18)	0.22(0.21,0.23)	0.22(0.20,0.23)	0.22(0.19,0.26)	0.16^***^	0.003^***^
		Both	0.09(0.08,0.10)	0.12(0.11,0.13)	0.15(0.15,0.17)	0.19(0.18,0.20)	0.19(0.17,0.20)	0.19(0.18,0.22)	0.15^***^	0.002^***^
	Incidence									
		Female	3.00(2.84,3.17)	2.65(2.51,2.76)	2.13(2.02,2.22)	2.18(2.06,2.27)	2.27(2.12,2.38)	2.26(2.03,2.51)	2.11^***^	0.01^*^
		Male	3.61(3.41,3.81)	3.32(3.14,3.49)	2.95(2.76,3.11)	3.28(3.09,3.45)	3.66(3.47,3.82)	3.72(3.37,4.14)	3.40^***^	0.03^*^
		Both	3.30(3.14,3.43)	2.97(2.83,3.07)	2.52(2.38,2.63)	2.69(2.54,2.82)	2.91(2.75,3.03)	2.93(2.66,3.27)	2.46^***^	0.02^*^
LAC	Mortality									
		Female	3.23(3.03,3.40)	2.81(2.65,2.94)	2.29(2.14,2.39)	2.30(2.15,2.42)	2.43(2.27,2.54)	2.40(2.16,2.67)	2.41^***^	0.07^**^
		Male	3.84(3.61,4.05)	3.50(3.30,3.69)	3.16(2.96,3.35)	3.45(3.22,3.64)	3.88(3.66,4.06)	3.95(3.57,4.38)	3.28^***^	0.11^*^
		Both	3.52(3.33,3.67)	3.14(2.97,3.27)	2.70(2.53,2.82)	2.83(2.65,2.97)	3.09(2.92,3.22)	3.11(2.82,3.44)	2.86^***^	0.09^*^
	1-MIR									
		Female	0.00(0.00,0.00)	0.00(0.00,0.00)	0.00(0.00,0.00)	0.00(0.00,0.00)	0.00(0.00,0.00)	0.00(0.00,0.00)	–	–
		Male	0.00(0.00,0.00)	0.00(0.00,0.00)	0.00(0.00,0.00)	0.00(0.00,0.00)	0.00(0.00,0.00)	0.00(0.00,0.00)	–	–
		Both	0.00(0.00,0.00)	0.00(0.00,0.00)	0.00(0.00,0.00)	0.00(0.00,0.00)	0.00(0.00,0.00)	0.00(0.00,0.00)	–	–
	Incidence									
		Female	3.92(3.12,4.81)	3.68(3.19,4.20)	3.42(3.13,3.73)	3.87(3.55,4.23)	4.17(3.76,4.75)	4.10(3.44,4.97)	3.93^***^	0.01^**^
		Male	8.18(7.08,9.30)	7.78(7.05,8.58)	7.79(7.27,8.33)	8.68(8.03,9.42)	8.93(7.84,10.22)	8.40(6.60,10.63)	6.46^***^	0.03^**^
		Both	6.08(5.28,6.81)	5.76(5.24,6.25)	5.64(5.29,5.99)	6.31(5.91,6.81)	6.59(5.93,7.42)	6.29(5.13,7.71)	5.30^***^	0.02^**^
NAME	Mortality									
		Female	4.13(3.28,5.12)	3.83(3.27,4.43)	3.61(3.28,3.93)	3.87(3.51,4.24)	3.95(3.55,4.45)	3.69(3.10,4.51)	4.30^***^	−0.001^**^
		Male	8.62(7.48,9.79)	8.18(7.39,9.08)	8.41(7.76,9.07)	8.67(8.00,9.38)	9.16(8.12,10.28)	8.63(6.81,10.83)	7.06^***^	−0.003^**^
		Both	6.39(5.55,7.19)	6.03(5.47,6.55)	6.04(5.62,6.42)	6.31(5.90,6.74)	6.59(5.99,7.31)	6.20(5.06,7.62)	5.79^***^	−0.003^**^
	1-MIR									
		Female	0.00(0.00,0.00)	0.00(0.00,0.00)	0.00(0.00,0.00)	0.04(0.05,0.04)	0.09(0.09,0.10)	0.13(0.13,0.12)	−0.007^***^	0.001^**^
		Male	0.00(0.00,0.00)	0.00(0.00,0.00)	0.00(0.00,0.00)	0.02(0.02,0.03)	0.00(0.00,0.02)	0.00(0.00,0.00)	0.004^***^	0.007^**^
		Both	0.00(0.00,0.00)	0.00(0.00,0.00)	0.00(0.00,0.00)	0.03(0.03,0.04)	0.03(0.02,0.04)	0.04(0.04,0.04)	−0.005^*^	0.003^*^
	Incidence									
		Female	1.96(1.54,2.45)	2.01(1.58,2.44)	2.05(1.65,2.43)	2.00(1.63,2.34)	1.98(1.67,2.29)	1.95(1.54,2.47)	–	–
		Male	3.29(2.64,3.91)	3.38(2.75,3.88)	3.52(2.96,3.91)	3.53(3.04,3.87)	3.45(3.13,3.76)	3.38(2.86,3.99)	–	–
		Both	2.66(2.20,3.08)	2.72(2.25,3.09)	2.79(2.37,3.11)	2.77(2.37,3.04)	2.70(2.42,2.94)	2.66(2.30,3.05)	–	–
SA^a^	Mortality									
		Female	2.13(1.66,2.70)	2.18(1.74,2.67)	2.25(1.80,2.66)	2.22(1.82,2.59)	2.08(1.74,2.44)	2.09(1.61,2.67)	–	–
		Male	3.46(2.78,4.07)	3.61(2.93,4.13)	3.74(3.13,4.19)	3.78(3.27,4.18)	3.59(3.28,3.92)	3.56(2.98,4.18)	–	–
		Both	2.82(2.33,3.27)	2.92(2.44,3.32)	3.01(2.54,3.35)	3.00(2.59,3.30)	2.82(2.56,3.09)	2.81(2.43,3.24)	–	–
	1-MIR									
		Female	0.00(0.00,0.00)	0.00(0.00,0.00)	0.00(0.00,0.00)	0.00(0.00,0.00)	0.00(0.00,0.00)	0.00(0.00,0.00)	–	–
		Male	0.00(0.00,0.01)	0.00(0.00,0.00)	0.00(0.00,0.00)	0.00(0.00,0.00)	0.00(0.00,0.00)	0.00(0.00,0.00)	–	–
		Both	0.00(0.00,0.00)	0.00(0.00,0.00)	0.00(0.00,0.00)	0.00(0.00,0.00)	0.00(0.00,0.00)	0.00(0.00,0.00)	–	–
	Incidence									
		Female	12.17(10.133,14.70)	12.18(10.91,13.77)	8.39(7.78,9.18)	5.17(4.79,5.58)	4.75(4.30,5.20)	4.75(3.97,5.61)	6.11^***^	−0.05^***^
		Male	29.69(24.58,35.49)	33.06(30.15,36.51)	23.46(21.73,25.32)	13.61(12.66,14.74)	13.85(12.57,15.34)	14.66(12.12,17.59)	12.63^***^	−0.10^*^
		Both	20.85(17.91,24.30)	22.50(20.94,24.41)	15.77(14.85,16.83)	9.27(8.71,9.98)	9.14(8.44,9.89)	9.52(8.19,11.01)	9.35^***^	−0.09^*^
SAEAO	Mortality									
		Female	12.70(10.53,15.36)	12.69(11.24,14.47)	8.10(7.25,9.15)	5.36(4.92,5.82)	4.73(4.27,5.22)	4.67(3.93,5.52)	6.38^***^	−0.07^***^
		Male	29.87(24.72,35.64)	33.42(30.05,37.20)	21.42(19.21,23.77)	13.61(12.49,14.79)	12.89(11.35,14.40)	13.40(11.17,15.84)	13.05^***^	−0.05^***^
		Both	21.13(18.37,24.44)	22.84(20.90,24.88)	14.56(13.37,15.89)	9.31(8.67,9.97)	8.61(7.84,9.43)	8.82(7.65,10.09)	9.60^***^	−0.05^*^
	1-MIR									
		Female	0.00(0.00,0.00)	0.00(0.00,0.00)	0.06(0.04,0.08)	0.00(0.00,0.00)	0.02(0.01,0.03)	0.03(0.02,0.04)	0.006	–
		Male	0.03(0.01,0.05)	0.02(0.01,0.04)	0.15(0.12,0.18)	0.04(0.03,0.06)	0.13(0.10,0.15)	0.11(0.09,0.12)	0.01	–
		Both	0.02(0.01,0.04)	0.03(0.01,0.05)	0.11(0.08,0.13)	0.03(0.02,0.05)	0.10(0.08,0.12)	0.09(0.08,0.10)	0.01	–
	Incidence									
		Female	3.31(2.83,3.95)	3.40(2.94,3.93)	3.37(2.94,3.84)	3.17(2.78,3.54)	3.06(2.69,3.43)	2.90(2.52,3.31)	3.73^*^	−0.002^*^
		Male	5.61(4.57,7.59)	6.68(5.99,7.60)	6.88(6.19,7.66)	6.34(5.73,7.00)	6.05(5.47,6.67)	5.75(5.09,6.46)	8.14^***^	−0.03^*^
		Both	4.43(3.77,5.54)	4.99(4.49,5.52)	5.05(4.57,5.59)	4.67(4.24,5.11)	4.45(4.05,4.89)	4.22(3.77,4.71)	5.90^***^	−0.01^*^
SSA	Mortality									
		Female	3.54(3.05,4.22)	3.63(3.16,4.17)	3.55(3.07,4.05)	3.37(2.95,3.76)	3.30(2.89,3.70)	3.13(2.72,3.59)	3.85^***^	−0.02^***^
		Male	6.01(4.91,8.14)	7.29(6.55,8.28)	7.41(6.58,8.32)	6.80(6.08,7.54)	6.54(5.88,7.20)	6.19(5.46,6.91)	10.56^***^	−0.05^*^
		Both	4.73(4.01,5.93)	5.38(4.83,5.96)	5.38(4.83,5.99)	4.98(4.49,5.48)	4.80(4.35,5.26)	4.53(4.03,5.04)	5.95^***^	−0.03^*^
	1-MIR									
		Female	0.00(0.00,0.00)	0.00(0.00,0.00)	0.00(0.00,0.00)	0.00(0.00,0.00)	0.00(0.00,0.00)	0.00(0.00,0.00)	–	–
		Male	0.00(0.00,0.00)	0.00(0.00,0.00)	0.00(0.00,0.00)	0.00(0.00,0.00)	0.00(0.00,0.00)	0.00(0.00,0.00)	–	–
		Both	0.00(0.00,0.00)	0.00(0.00,0.00)	0.00(0.00,0.00)	0.00(0.00,0.00)	0.00(0.00,0.00)	0.00(0.00,0.00)	–	–
	Incidence									
		Female	5.22(4.62,5.96)	5.55(5.14,6.04)	4.69(4.40,4.95)	3.80(3.56,3.98)	3.66(3.40,3.87)	3.63(3.23,4.05)	3.99^***^	−0. 03^***^
		Male	13.07(11.42,14.87)	15.02(14.06,16.16)	12.63(12.02,13.26)	9.60(9.18,10.06)	9.59(9.07,10.11)	9.71(8.69,10.84)	7.91^***^	−0. 08^*^
		Both	8.98(8.10,9.97)	10.09(9.56,10.70)	8.47(8.10,8.84)	6.55(6.25,6.82)	6.47(6.11,6.77)	6.51(5.95,7.16)	5.83^***^	−0. 07^*^
Global	Mortality									
		Female	5.33(4.67,6.09)	5.59(5.15,6.11)	4.47(4.15,4.80)	3.72(3.44,3.91)	3.51(3.22,3.74)	3.46(3.08,3.83)	4.17^***^	−0. 02^***^
		Male	12.90(11.30,14.67)	14.76(13.65,15.99)	11.48(10.70,12.29)	9.00(8.55,9.45)	8.66(8.10,9.22)	8.73(7.88,9.60)	8.14^***^	−0. 07^*^
		Both	8.93(8.09,9.90)	9.95(9.32,10.60)	7.78(7.35,8.20)	6.20(5.87,6.46)	5.93(5.59,6.24)	5.95(5.44,6.44)	6.03^***^	−0. 06^*^
	1-MIR									
		Female	0.00(0.00,0.00)	0.01(0.00,0.03)	0.07(0.05,0.09)	0.04(0.03,0.06)	0.05(0.05,0.03)	0.04(0.02,0.05)	0.006	0.01^*^
		Male	0.04(0.02,0.05)	0.05(0.04,0.07)	0.11(0.09,0.13)	0.09(0.08,0.11)	0.11(0.10,0.13)	0.11(0.10,0.13)	0.01	0.01^*^
		Both	0.02(0.01,0.03)	0.04(0.03,0.07)	0.09(0.08,0.11)	0.07(0.06,0.09)	0.09(0.06,0.10)	0.09(0.07,0.11)	0.01	0.01^*^

Data in parentheses are 95% uncertainty intervals

*CEEECA* Central Europe, Eastern Europe, and Central Asia, *HI* High Income, *LAC* Latin America and Caribbean, *NAME* North Africa and Middle East, *SA* South Asia, *SAEAO* Southeast Asia, East Asia, and Oceania, *SSA* Sub-Saharan Africa, *MIR *Mortality-to-incidence ratio, *LGCM *Latent growth curve model

^*^ *p*<0.05, ^**^ *p*>0.05, ^***^ *p*<0.001

^a^Not fitted LGCM, due to inadequate number of countries

In terms of liver cancer mortality, the age-standardized mortality rates (ASMRs) were higher among men than women in all regions of the world. In addition, the LGCMs identified that initial values were much higher among males compared to females in 1990. The countries in SAEAO experienced the highest overall mean of initial LC rate for males (13.05) and females (6.05); and the countries in LAC with 3.28 (for males) and HI with 2.21 (for females) had the lowest of initial rate of LC at the starting point of the study. As such, in men and women, the most significant changes in mortality rates were detected in CEEECA (with slopes of 0.15 and 0.11, respectively), whereas North Africa and Middle East (NAME) was the area with the lowest changes in mortality due to LC, followed by SA over the period of analysis. Figure 1 shows that ASMR trends varied among the different regions for males and females across the reporting period. Notable declines were evident in the ASMRs of LAC (from 1993 to 2000) and SAEAO (from 1997 to 2006) for males. Subsequently, the largest increases in ASMRs were observed in LAC (with a peak in 2017), SSA (with a peak in 1998), and HI (with a peak in 2000) among males.

The largest [1-MIR] for each gender recorded in HI countries and the least [1-MIR] recorded in CEEECA, LAC, NAME, SA, and SSA. Accordingly, countries in HI recorded the highest initial values of [1-MIRs] for both men and women in 1990. It is worth noting that during the study period, the average rates of change in HI showed that [1-MIRs] had upward trends among females and males (Table 1). Figure 1 exhibited that each continent has its unique pattern in terms of [1-MIR] among both males and females. For both genders, SAEAO countries have not experienced systematic trends over time. In males, CEEECA, followed by SA and NAME had also not regular temporal patterns; in LAC and SSA, the trends of [1-MIR] stayed steady (flat) for them over time. Furthermore, the increment in [1-MIR] rate was more prominent during 1990–1993 and 1999–2000 in males of HI. Among women, static trends in [1-MIR] was observed in LAC, CEEECA, SA, and SSA over the entire reporting period, while there was a substantial rise in [1-MIR] in NAME from 2010 to 2019.

With regards to the last row of Table 1 which gives information on the intercepts and slopes of the three metrics separately for 184 countries men and women, the overall mean intercept of LC ASIR and ASMR among males were, on average, higher than females, 7.91 vs. 3.99 and 8.14 vs. 4.17, respectively in 1990. As well, we found statistically significant changes in trends of ASIR, ASMR, and [1-MIR] both in males and females with negative slopes, which disclosed trivial declines in growth trajectories between 1990 and 2019.

**Fig. 2:**
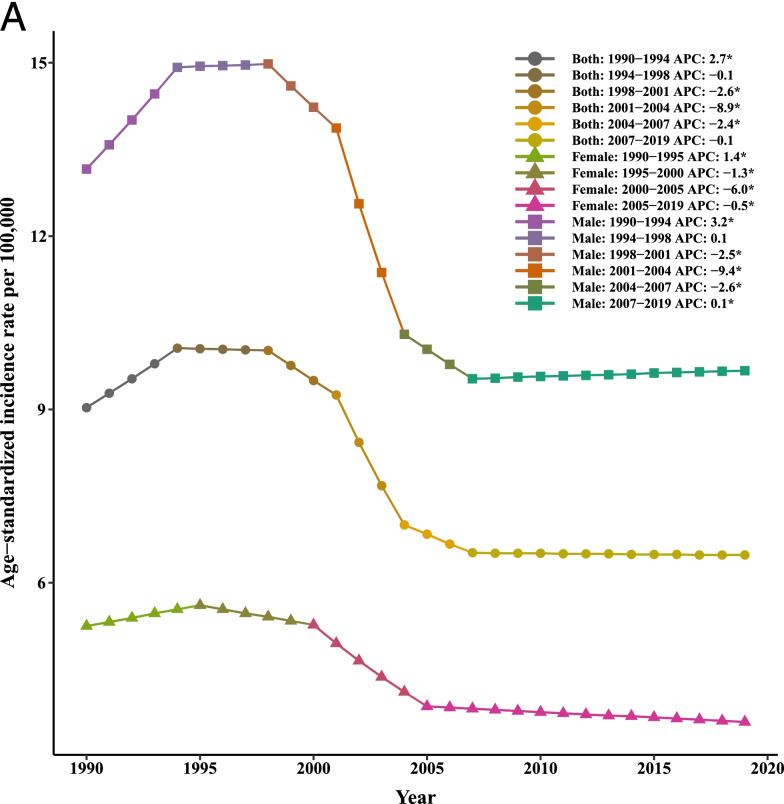

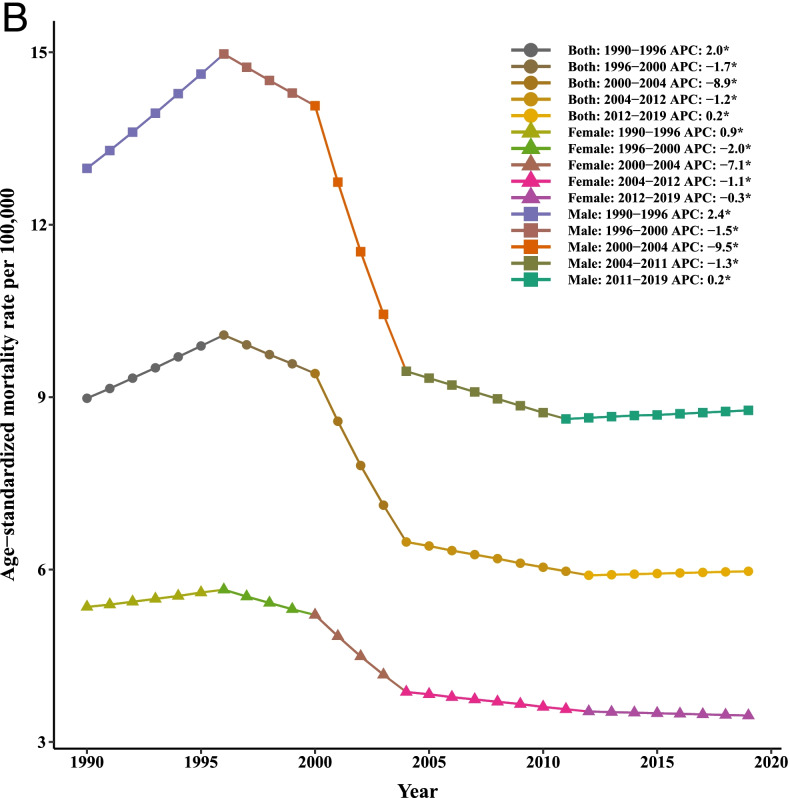

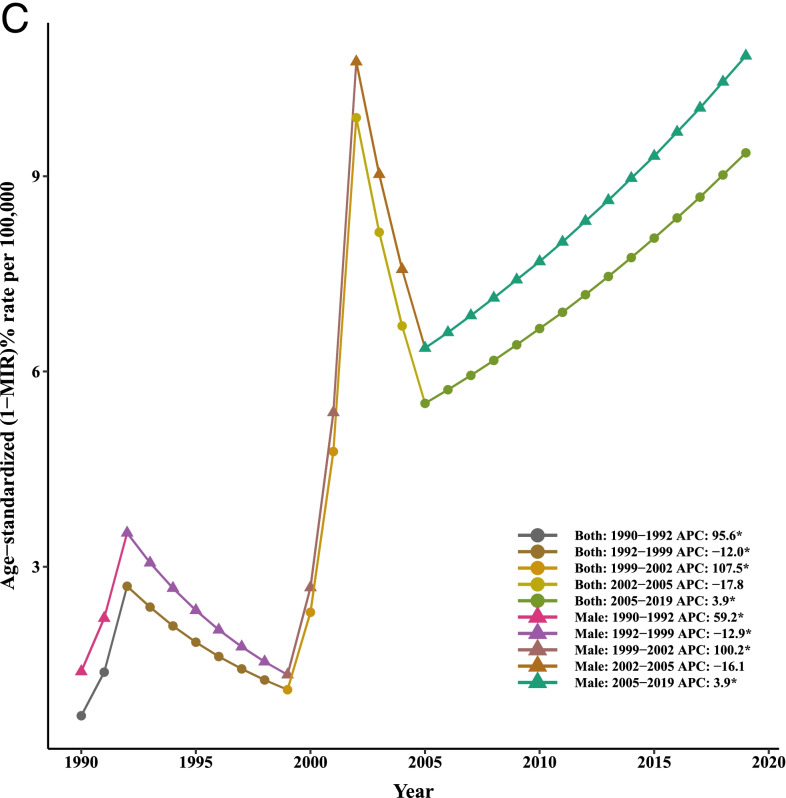
Joinpoint regression analyses of trends in mean trends of liver cancer (**A**) incidence, (**B**) mortality, and (**C**) [1-MIR] per 100,000 by gender worldwide, 1990–2019

Our joinpoint analysis indicated a significant downward trend of incidence and death in males and females between 1990 and 2019. Accordingly, the AAPCs of incidence (− 1.10, 95% CI:-1.40,-0.90) and mortality (− 1.40, 95% CI:-1.50,-1.30) were significantly decreased for both genders over time. Among males, changes of the [1-MIR] disclosed an upward trend; however. However, no results were obtained for females due to the lack of MIR data for them from 1990 to 1999 and very close to zero in other years. Therefore, the APC could not be calculated using joinpoint software. In total population, the AAPC of [1-MIR] was positive and statistically significant (4.90, 95% CI: 1.70, 8.10) in the period 1990–2019 (Table 2 and Fig. 2).

**Table 2:** Joinpoint trend analysis of liver cancer age-standardized incidence, mortality, and 1-MIR rates worldwide by sex, 1990–2019

Measure	Trend	Both		Male		Female	
		Year	APC (95%CI)	Year	APC (95%CI)	Year	APC (95%CI)
Incidence							
	Trend 1	1990–1994	2.70*(2.30,3.10)	1990–1994	3.20*(2.80,3.60)	1990–1995	1.30*(1.20,1.50)
	Trend 2	1994–1998	−0.10(−0.70,0.50)	1994–1998	0.10(−0.60,0.70)	1995–2000	−1.30*(−1.50,-1)
	Trend 3	1998–2001	−2.60*(−3.80,-1.40)	1998–2001	−2.50*(−3.80,-1.30)	2000–2005	−6.0*(−6.20,-5.70)
	Trend 4	2001–2004	−8.90*(−10,-7.70)	2001–2004	−9.40*(−10.60,-8.30)	2005–2015	− 0.60*(− 0.70,-0.50)
	Trend 5	2004–2007	−2.40*(−3.60,-1.20)	2004–2007	−2.60*(−3.80,-1.30)	2015–2019	−0.10(− 0.40,0.10)
	Trend 6	2007–2019	0(−0.10,0)	2007–2019	0.10*(0,0.20)		
	AAPC	1990–2019	−1.10*(−1.40,-0.90)	1990–2019	− 1.10*(− 1.30,-0.80)	1990–2019	− 1.30*(− 1.40,-1.20)
Mortality							
	Trend 1	1990–1996	1.90*(1.70,2.20)	1990–1996	2.40*(2.10,2.70)	1990–1996	0.90*(0.80,1.10)
	Trend 2	1996–2000	−1.70*(− 2.30,-1.10)	1996–2000	− 1.50*(− 2.30,-0.80)	1996–2000	− 2.0*(− 2.40,-1.60)
	Trend 3	2000–2004	−8.90*(− 9.50,-8.30)	2000–2004	− 9.40*(− 10.10,-8.80)	2000–2004	−7.20*(−7.60,-6.80)
	Trend 4	2004–2012	− 1.20*(− 1.30,-1)	2004–2011	− 1.30*(− 1.60,− 1.10)	2004–2012	-1.10*(− 1.30,-1)
	Trend 5	2012–2019	0.20*(0,0.30)	2011–2019	0.20*(0,0.40)	2012–2019	− 0.30*(− 0.40,-0.20)
	AAPC	1990–2019	− 1.40*(− 1.50,− 1.30)	1990–2019	-1.30*(− 1.50,-1.20)	1990–2019	−1.50*(− 1.60,-1.40)
1-MIR							
	Trend 1	1990–1992	34.30*(10.80,62.70)	1990–1992	22.90*(3.60,45.70)	–	–
	Trend 2	1992–1999	−7.50*(− 10.40,-4.40)	1992–1999	− 7.0*(− 9.60,-4.30)	–	–
	Trend 3	1999–2002	65.50*(36.50,100.50)	1999–2002	54.80*(30.60,83.60)	–	–
	Trend 4	2002–2005	−16.60(− 31.10,1.10)	2002–2005	−13.90(− 27.40,2.10)	–	–
	Trend 5	2005–2012	5.70*(2.30,9.20)	2005–2012	5.30*(2.30,8.30)	–	–
	Trend 6	2012–2019	−0.40(− 2.90,2.20)	2012–2019	− 0.10(− 2.40,2.20)	–	–
	AAPC	1990–2019	4.90*(1.70,8.10)	1990–2019	3.90*(1.20,6.80)	–	–

*APC* Annual Percentage Change, *AAPC* Average Annual Percent Change, *CI *Confidence Interval, *MIR *Mortality-to-incidence Ratio

*Significantly different from 0 at alpha = 0.05 (*p* < 0.05). There are 1+(number of trend) joinpoints for each model

**Fig. 3:**
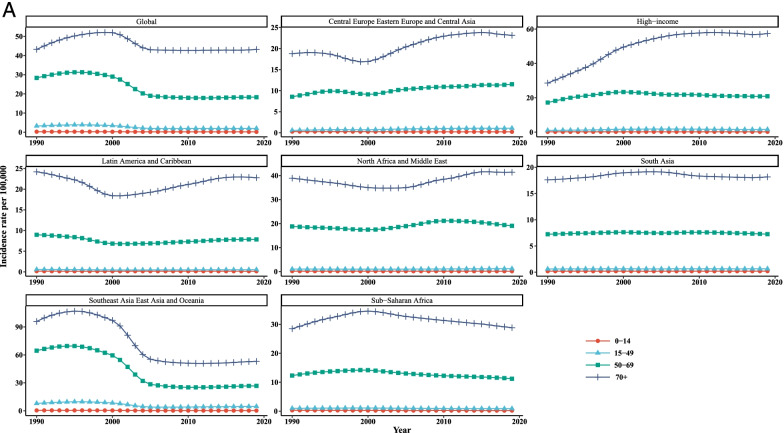

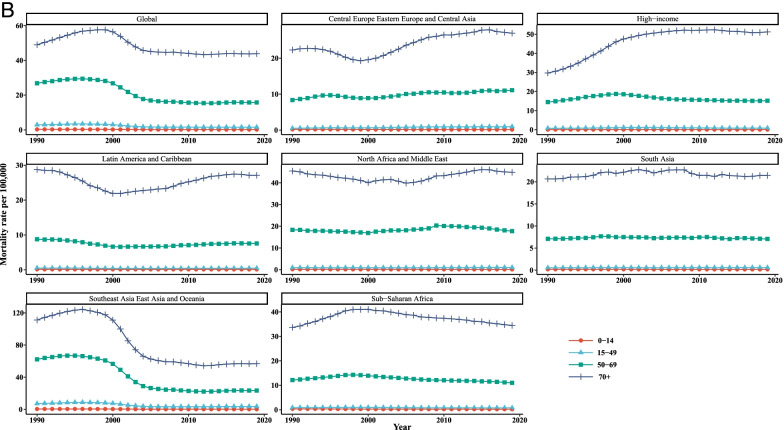

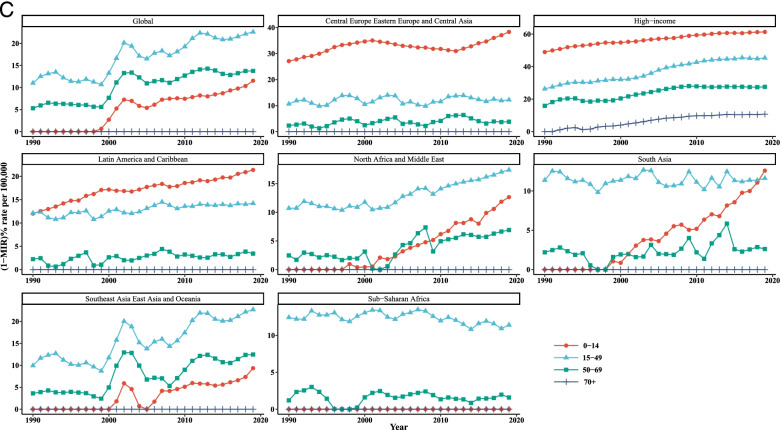
Mean temporal trends in liver cancer (**A**) incidence, (**B**) mortality, and (**C**) [1-MIR] per 100,000 by age group in each super-region, 1990–2019

Overall, it is evident from Table 3 and Fig. 3 that the incidence, mortality, and [1-MIR] rates vary across the age groups in all super-regions such that people aged 70+ years experienced higher rates than that of the other age groups throughout the study period. Taking the time trends into consideration, it was obvious that the incidence and mortality rates in the age groups of 0–14 and 15–49 years were quite similar and almost overlapped among the super-regions over the whole study period. According to the findings of LGCM, the highest initial values of LC incidence and mortality rates were observed for the age group of older than 70 years in SAEAO countries, 48.09/100,000 and 56.50/100,000, respectively. As well, the lowest starting points in 1990 for incidence and mortality were related to NAME (0.12/100,000) and HI (0.08/100,000) courtiers among the youngest group (0 to 14 years), respectively. Moreover, the 50–69 and 70+ age groups recorded the greatest and lowest initial value of [1-MIR] (10.45/100,000) in HI, respectively at the beginning of the study. Additionally, the average rate of incidence and mortality changes were the highest in CEEECA with slopes of 0.90 and 1.06, respectively, for the people 70+ years of age; and the lowest were occurred in SSA for the age groups 0–14 years and 15–49 years. Countries of HI had the highest average rate of change in [1-MIR] among persons aged 50–69 years. As can be seen in Fig. 1, HI countries had the most drastic rises in incidence and mortality in people aged older than 70 since 1990 to 2019. The decreases of incidence and mortality were substantial in SAEAO for both 50–69 and 70+ years from 1997 to 2007. Nonetheless, in countries of SA, followed by NAME, the incidence and mortality had slow and moderate changes in LC incidence and mortality trends for total population in each age group over the 30-year study period. Moreover, at a glance, subjects aged younger than 14 years had sharp increasing trends of [1-MIR] in NAME and SA countries compared to their counterparts from 2000 to 2019. The last row of Table 2 revealed that the incidence and mortality had downward trends for all age groups worldwide over time. In contrast, persons aged 0–14, 15–49, and 50–69 years experienced significant incrementing trends with gentle slopes during the study period. The incidence and mortality tended to rise in both subjects 50–69 and 70+ years of age up to the mid-1999 and fallen sharply during 2000–2005, but it displayed no further changes in the period between 2006 and 2019. Moreover, a stable trend was observed in [1-MIR] among people aged 70+ years during the past three decades.

**Table 3:** Age specific incidence, mortality, and 1-MIR rates per 100,000 people as well as LGCM results stratified by age group and super- region, 1990–2019

Super- region	Metric	Age group	Year						LGCM estimate	
			1990	1996	2002	2008	2014	2019	Intercept	Slope
	Incidence									
		0–14	0.28(0.22,0.38)	0.28(0.22,0.39)	0.24(0.18,0.36)	0.22(0.14,0.36)	0.22(0.13,0.38)	0.23(0.13,0.41)	0.22^*^	−0.002^*^
		15–49	0.57(0.54,0.60)	0.74(0.70,0.78)	0.78(0.76,0.81)	1.00(0.96,1.04)	1.07(1.02,1.12)	1.09(0.96,1.23)	0.89^*^	0.03^*^
		50–69	8.55(8.31,8.81)	9.88(9.58,10.21)	9.51(9.29,9.72)	10.75(10.44,11.05)	11.19(10.87,11.55)	11.53(10.41,12.70)	20.80^*^	0.07^*^
		> 70	18.78(17.82,19.39)	18.24(17.44,18.79)	18.05(17.28,18.46)	22.12(21.12,22.76)	23.73(22.48,24.52)	23.14(21.06,25.14)	32.38^*^	0.90^*^
CEEECA	Mortality									
		0–14	0.20(0.17,0.22)	0.19(0.17,0.21)	0.16(0.14,0.20)	0.15(0.11,0.21)	0.15(0.11,0.22)	0.14(0.09,0.22)	0.15^*^	−0.002^*^
		15–49	0.51(0.49,0.54)	0.65(0.62,0.68)	0.68(0.65,0.71)	0.90(0.86,0.95)	0.93(0.88,0.98)	0.96(0.85,1.09)	0.82^***^	0.02^*^
		50–69	8.35(8.12,8.60)	9.53(9.28,9.81)	9.12(8.89,9.38)	10.51(10.18,10.83)	10.62(10.31,10.98)	11.09(10.00,12.26)	20.53^*^	0.04^*^
		> 70	22.28(21.10,23.01)	21.11(20.15,21.72)	20.72(19.78,21.30)	25.82(24.57,26.59)	27.28(25.70,28.23)	26.95(24.34,29.39)	38.21^*^	1.06^*^
	1-MIR									
		0–14	0.27(0.21,0.41)	0.33(0.25,0.45)	0.35(0.24,0.44)	0.32(0.21,0.41)	0.33(0.18,0.42)	0.38(0.26,0.47)	0.30^***^	0.002^*^
		15–49	0.11(0.11,0.11)	0.12(0.11,0.13)	0.13(0.13,0.12)	0.10(0.11,0.09)	0.13(0.13,0.13)	0.12(0.12,0.12)	0.10^*^	0.002^*^
		50–69	0.02(0.02,0.02)	0.04(0.03,0.04)	0.04(0.04,0.04)	0.02(0.02,0.02)	0.05(0.05,0.05)	0.04(0.04,0.03)	0.02^*^	0.001^*^
		> 70	0.00(0.00,0.00)	0.00(0.00,0.00)	0.00(0.00,0.00)	0.00(0.00,0.00)	0.00(0.00,0.00)	0.00(0.00,0.00)	–	–
	Incidence									
		0–14	0.17(0.14,0.21)	0.18(0.15,0.22)	0.19(0.16,0.23)	0.20(0.17,0.25)	0.20(0.17,0.25)	0.20(0.16,0.26)	0.16^***^	0.001^***^
		15–49	1.04(0.99,1.10)	1.31(1.28,1.35)	1.75(1.71,1.81)	1.78(1.72,1.84)	1.57(1.51,1.64)	1.57(1.39,1.77)	0.76^***^	0.02^***^
		50–69	17.22(16.85,17.60)	21.66(21.17,22.14)	23.00(22.55,23.41)	21.82(21.30,22.27)	21.02(20.50,21.47)	20.94(18.92,23.31)	10.45^***^	0.09^***^
		> 70	28.57(26.94,29.47)	39.76(36.83,41.28)	52.17(47.82,54.26)	57.20(51.25,60.24)	57.67(50.69,61.44)	57.38(49.26,63.45)	30.70^***^	0.63^***^
HI	Mortality									
		0–14	0.09(0.08,0.10)	0.08(0.08,0.10)	0.09(0.08,0.09)	0.08(0.08,0.09)	0.08(0.07,0.09)	0.08(0.07,0.09)	0.08^***^	0.01^*^
		15–49	0.76(0.72,0.82)	0.91(0.89,0.95)	1.17(1.13,1.22)	1.05(1.01,1.10)	0.87(0.83,0.90)	0.86(0.79,0.94)	0.58^***^	0.01^*^
		50–69	14.49(14.19,14.78)	17.65(17.28,17.99)	17.76(17.42,18.09)	15.80(15.41,16.14)	15.21(14.87,15.49)	15.18(14.29,15.86)	10.45^***^	0.09^*^
		> 70	29.71(27.99,30.67)	39.15(36.28,40.59)	49.36(45.03,51.39)	52.13(46.38,54.94)	51.58(45.19,54.79)	51.22(44.59,54.84)	33.37^***^	0.46^***^
	1-MIR									
		0–14	0.49(0.45,0.52)	0.53(0.49,0.57)	0.56(0.53,0.60)	0.58(0.56,0.62)	0.61(0.58,0.65)	0.61(0.57,0.66)	0.49^***^	0.004^*^
		15–49	0.26(0.27,0.26)	0.30(0.31,0.30)	0.33(0.34,0.33)	0.41(0.41,0.41)	0.45(0.45,0.45)	0.45(0.43,0.47)	0.24^***^	0.01^*^
		50–69	0.16(0.16,0.16)	0.19(0.18,0.19)	0.23(0.23,0.23)	0.28(0.28,0.28)	0.28(0.27,0.28)	0.28(0.24,0.32)	10.45^***^	0.09^*^
		> 70	0.00(0.00,0.00)	0.02(0.01,0.02)	0.05(0.06,0.05)	0.09(0.09,0.09)	0.11(0.11,0.11)	0.11(0.09,0.14)	0.003^***^	0.001^***^
	Incidence									
		0–14	0.16(0.13,0.20)	0.15(0.12,0.19)	0.15(0.12,0.19)	0.16(0.12,0.20)	0.15(0.12,0.20)	0.15(0.11,0.19)	0.13^*^	0.01^**^
		15–49	0.57(0.55,0.60)	0.54(0.52,0.56)	0.46(0.44,0.48)	0.48(0.46,0.50)	0.50(0.47,0.52)	0.53(0.46,0.60)	0.53^***^	0.004^**^
		50–69	8.96(8.58,9.34)	8.15(7.86,8.46)	6.78(6.50,7.05)	7.15(6.86,7.46)	7.70(7.40,8.01)	7.84(7.09,8.76)	8.72^***^	−0.03^***^
		> 70	24.21(22.41,25.46)	21.64(20.04,22.70)	18.52(16.91,19.59)	20.40(18.62,21.49)	22.62(20.70,23.85)	22.78(20.19,25.18)	23.19^***^	0.28^*^
LAC	Mortality									
		0–14	0.14(0.12,0.16)	0.13(0.11,0.14)	0.12(0.11,0.14)	0.13(0.11,0.15)	0.12(0.10,0.14)	0.12(0.09,0.14)	0.11^*^	0.01^**^
		15–49	0.50(0.48,0.52)	0.47(0.45,0.49)	0.40(0.39,0.42)	0.41(0.40,0.43)	0.43(0.41,0.45)	0.45(0.40,0.51)	0.47^***^	0.003^**^
		50–69	8.76(8.38,9.15)	7.91(7.61,8.22)	6.64(6.35,6.93)	6.87(6.58,7.20)	7.45(7.17,7.75)	7.57(6.90,8.44)	6.97^***^	0.06^***^
		> 70	28.75(26.60,30.26)	25.48(23.41,26.84)	22.23(20.26,23.54)	23.97(21.71,25.45)	26.99(24.65,28.42)	27.11(24.15,29.92)	20.116^***^	0.28^*^
	1-MIR									
		0–14	0.12(0.07,0.19)	0.15(0.08,0.24)	0.17(0.11,0.27)	0.18(0.12,0.26)	0.19(0.15,0.28)	0.21(0.18,0.26)	0.21^*^	0.003^**^
		15–49	0.12(0.12,0.12)	0.12(0.12,0.12)	0.12(0.12,0.12)	0.14(0.14,0.13)	0.14(0.14,0.14)	0.14(0.14,0.15)	0.11^*^	0.001^**^
		50–69	0.02(0.02,0.02)	0.03(0.03,0.03)	0.02(0.02,0.02)	0.04(0.04,0.03)	0.03(0.03,0.03)	0.03(0.03,0.04)	0.02^*^	0.001^*^
		> 70	0.00(0.00,0.00)	0.00(0.00,0.00)	0.00(0.00,0.00)	0.00(0.00,0.00)	0.00(0.00,0.00)	0.00(0.00,0.00)	–	–
	Incidence									
		0–14	0.18(0.11,0.28)	0.16(0.11,0.23)	0.16(0.11,0.22)	0.16(0.11,0.21)	0.15(0.11,0.21)	0.14(0.10,0.20)	0.12^***^	−0.01^**^
		15–49	1.02(0.86,1.23)	1.02(0.88,1.20)	1.01(0.90,1.14)	1.06(0.95,1.21)	1.16(1.00,1.36)	1.21(0.93,1.56)	0.80^***^	0.003^**^
		50–69	18.79(16.18,21.53)	18.01(16.06,19.91)	17.78(16.35,19.13)	20.53(18.67,23.07)	20.73(17.88,24.49)	19.05(14.66,24.73)	14.92^***^	−0.02^**^
		> 70	38.91(31.72,45.67)	36.74(32.14,40.97)	34.76(31.71,37.88)	37.16(34.30,39.94)	41.14(37.39,45.27)	41.38(34.93,49.23)	37.34^***^	0.27^**^
NAME	Mortality									
		0–14	0.19(0.13,0.29)	0.16(0.12,0.22)	0.15(0.12,0.20)	0.15(0.11,0.20)	0.14(0.11,0.18)	0.12(0.09,0.17)	0.12^***^	−0.001^**^
		15–49	0.92(0.77,1.10)	0.91(0.77,1.08)	0.90(0.79,1.04)	0.91(0.80,1.04)	0.98(0.83,1.16)	1.00(0.75,1.31)	0.72^***^	0.001^**^
		50–69	18.33(15.76,20.99)	17.61(15.62,19.62)	17.79(16.17,19.39)	19.02(17.33,21.05)	19.48(16.79,22.70)	17.73(13.56,23.21)	14.69^***^	−0.05^**^
		> 70	45.40(37.11,53.20)	42.42(36.69,47.35)	41.37(37.45,45.03)	41.77(38.26,45.43)	45.60(41.27,49.93)	44.80(37.62,53.57)	45.17^***^	0.06^**^
	1-MIR									
		0–14	0.00(0.00,0.00)	0.00(0.00,0.03)	0.02(0.00,0.08)	0.05(0.00,0.08)	0.09(0.01,0.13)	0.13(0.08,0.12)	0.08^***^	0.008^**^
		15–49	0.11(0.11,0.10)	0.11(0.12,0.10)	0.11(0.12,0.09)	0.14(0.15,0.14)	0.16(0.17,0.15)	0.17(0.19,0.16)	0.11^***^	0.004^**^
		50–69	0.02(0.03,0.03)	0.02(0.03,0.01)	0.00(0.01,0.00)	0.07(0.07,0.09)	0.06(0.06,0.07)	0.07(0.08,0.06)	0.02^***^	0.003^**^
		> 70	0.00(0.00,0.00)	0.00(0.00,0.00)	0.00(0.00,0.00)	0.00(0.00,0.00)	0.00(0.00,0.00)	0.00(0.00,0.00)	–	–
SA^a^	Incidence									
		0–14	0.18(0.14,0.25)	0.19(0.15,0.26)	0.19(0.15,0.26)	0.19(0.15,0.27)	0.19(0.15,0.27)	0.20(0.15,0.30)	–	–
		15–49	0.61(0.53,0.70)	0.64(0.56,0.71)	0.64(0.57,0.70)	0.65(0.58,0.72)	0.64(0.57,0.71)	0.65(0.56,0.76)	–	–
		50–69	7.24(6.04,8.45)	7.48(6.28,8.48)	7.55(6.35,8.41)	7.57(6.46,8.43)	7.49(6.75,8.14)	7.25(6.14,8.48)	–	–
		> 70	17.61(13.90,20.88)	18.21(14.50,21.36)	19.09(15.74,21.69)	18.63(15.64,20.88)	18.14(15.81,20.05)	18.17(15.63,20.82)	–	–
	Mortality									
		0–14	0.20(0.16,0.24)	0.20(0.16,0.24)	0.18(0.15,0.22)	0.17(0.14,0.21)	0.17(0.14,0.21)	0.17(0.14,0.22)	–	–
		15–49	0.54(0.47,0.62)	0.57(0.50,0.64)	0.57(0.50,0.63)	0.58(0.52,0.64)	0.56(0.51,0.62)	0.58(0.49,0.67)	–	–
		50–69	7.08(5.88,8.16)	7.44(6.31,8.41)	7.43(6.21,8.35)	7.37(6.25,8.21)	7.05(6.34,7.74)	7.06(6.09,8.24)	–	–
		> 70	20.64(16.27,24.41)	21.47(17.07,25.10)	22.75(18.96,25.70)	22.68(19.43,25.21)	21.40(19.08,23.59)	21.43(18.45,24.75)	–	–
	1-MIR									
		0–14	0.00(0.00,0.02)	0.00(0.00,0.08)	0.03(0.00,0.15)	0.06(0.01,0.21)	0.08(0.03,0.22)	0.13(0.08,0.27)	–	–
		15–49	0.11(0.11,0.12)	0.11(0.11,0.10)	0.12(0.13,0.10)	0.11(0.10,0.11)	0.12(0.11,0.13)	0.12(0.12,0.11)	–	–
		50–69	0.02(0.03,0.03)	0.01(0.00,0.01)	0.02(0.02,0.01)	0.03(0.03,0.03)	0.06(0.06,0.05)	0.03(0.01,0.03)	–	–
		> 70	0.00(0.00,0.00)	0.00(0.00,0.00)	0.00(0.00,0.00)	0.00(0.00,0.00)	0.00(0.00,0.00)	0.00(0.00,0.00)	–	–
	Incidence									
		0–14	0.43(0.35,0.54)	0.46(0.39,0.54)	0.31(0.27,0.37)	0.21(0.18,0.27)	0.21(0.17,0.26)	0.20(0.16,0.25)	0.22^***^	−0.002^*^
		15–49	7.95(6.75,9.37)	9.73(8.96,10.61)	6.64(6.23,7.13)	3.94(3.66,4.34)	4.39(3.98,4.87)	4.65(3.87,5.57)	2.74^***^	0.003^**^
		50–69	64.55(54.53,77.31)	68.76(63.17,75.15)	47.12(44.09,50.66)	25.76(24.21,27.68)	25.66(23.59,28.04)	26.76(22.59,31.60)	29.44^***^	−0.22^*^
		> 70	95.94(83.43,110.85)	106.62(98.76,114.94)	81.10(75.96,86.32)	51.83(48.48,55.38)	51.16(47.22,54.69)	53.07(46.30,59.79)	48.09^***^	−0.17^*^
SAEAO	Mortality									
		0–14	0.47(0.38,0.61)	0.49(0.43,0.57)	0.29(0.25,0.35)	0.20(0.18,0.24)	0.19(0.17,0.23)	0.18(0.15,0.22)	0.26^***^	−0.003^***^
		15–49	7.16(6.03,8.45)	8.75(7.96,9.63)	5.31(4.86,5.87)	3.38(3.10,3.72)	3.49(3.07,3.94)	3.60(3.00,4.26)	2.41^***^	−0.002^**^
		50–69	62.21(52.55,74.29)	66.15(59.95,72.75)	41.03(37.32,45.50)	24.39(22.42,26.42)	22.69(20.37,25.13)	23.43(19.94,27.09)	28.74^***^	−0.24^*^
		> 70	111.15(97.85,127.62)	124.28(114.20,135.29)	85.31(78.54,92.30)	59.15(54.82,63.10)	55.57(51.05,59.80)	56.77(49.49,63.45)	56.50^***^	−0.22^*^
	1-MIR									
		0–14	0.00(0.00,0.00)	0.00(0.00,0.00)	0.06(0.05,0.07)	0.04(0.01,0.10)	0.05(0.03,0.11)	0.09(0.09,0.11)	0.06^*^	0.003^*^
		15–49	0.10(0.11,0.10)	0.10(0.11,0.09)	0.20(0.22,0.18)	0.14(0.15,0.14)	0.21(0.23,0.19)	0.23(0.23,0.23)	0.11^*^	0.001^***^
		50–69	0.04(0.04,0.04)	0.04(0.05,0.03)	0.13(0.15,0.10)	0.05(0.07,0.05)	0.12(0.14,0.10)	0.12(0.12,0.14)	0.03^*^	0.001^***^
		> 70	0.00(0.00,0.00)	0.00(0.00,0.00)	0.00(0.00,0.00)	0.00(0.00,0.00)	0.00(0.00,0.00)	0.00(0.00,0.00)	–	–
	Incidence									
		0–14	0.29(0.19,0.42)	0.26(0.18,0.37)	0.22(0.15,0.30)	0.21(0.15,0.29)	0.21(0.14,0.30)	0.19(0.12,0.28)	0.27^*^	−0.004^*^
		15–49	0.97(0.81,1.24)	1.06(0.93,1.21)	1.06(0.92,1.23)	0.94(0.82,1.07)	0.89(0.77,1.02)	0.89(0.74,1.05)	1.30^***^	−0.005^*^
		50–69	12.30(10.32,15.48)	13.84(12.22,15.56)	13.75(12.22,15.53)	12.51(11.10,13.94)	11.88(10.58,13.31)	11.20(9.74,12.85)	17.89^***^	−0.03^*^
		> 70	28.46(24.17,35.24)	32.75(29.50,36.24)	34.04(30.92,37.13)	31.85(29.36,34.63)	30.32(27.83,32.92)	28.83(26.17,31.56)	40.80^***^	−0.13^*^
SSA	Mortality									
		0–14	0.36(0.26,0.50)	0.32(0.24,0.43)	0.26(0.20,0.34)	0.25(0.19,0.32)	0.25(0.18,0.32)	0.22(0.15,0.31)	0.34^***^	−0.004^*^
		15–49	0.85(0.71,1.10)	0.92(0.81,1.05)	0.92(0.80,1.07)	0.81(0.71,0.94)	0.79(0.68,0.91)	0.78(0.65,0.93)	1.08^***^	−0.006^*^
		50–69	12.15(10.28,15.29)	13.84(12.30,15.51)	13.41(11.82,15.24)	12.21(10.78,13.62)	11.78(10.46,13.15)	11.02(9.57,12.58)	19.33^***^	−0.09^*^
		> 70	33.65(28.59,41.74)	39.24(35.32,43.47)	40.43(36.38,44.42)	37.76(34.66,41.08)	36.13(33.18,39.20)	34.46(31.28,37.67)	47.06^*^	−0.09^*^
	1-MIR									
		0–14	0.00(0.00,0.00)	0.00(0.00,0.00)	0.00(0.00,0.00)	0.00(0.00,0.00)	0.00(0.00,0.00)	0.00(0.00,0.00)	–	–
		15–49	0.12(0.12,0.12)	0.13(0.13,0.13)	0.13(0.13,0.13)	0.13(0.14,0.12)	0.11(0.11,0.11)	0.11(0.12,0.12)	0.13^*^	−0.001^**^
		50–69	0.01(0.00,0.01)	0.00(0.00,0.00)	0.02(0.03,0.02)	0.02(0.03,0.02)	0.01(0.01,0.01)	0.02(0.02,0.02)	0.01^*^	0.001^**^
		> 70	0.00(0.00,0.00)	0.00(0.00,0.00)	0.00(0.00,0.00)	0.00(0.00,0.00)	0.00(0.00,0.00)	0.00(0.00,0.00)	–	–
	Incidence									
		0–14	0.27(0.22,0.34)	0.27(0.23,0.33)	0.22(0.19,0.27)	0.19(0.16,0.24)	0.19(0.16,0.24)	0.19(0.15,0.24)	0.20^***^	−0.001^*^
		15–49	3.25(2.83,3.74)	3.88(3.61,4.17)	2.86(2.71,3.02)	1.94(1.84,2.07)	1.99(1.85,2.13)	1.99(1.75,2.26)	1.30^***^	0.008^*^
		50–69	28.35(25.27,32.11)	31.30(29.56,33.42)	25.14(24.08,26.32)	18.19(17.57,18.91)	17.99(17.17,18.87)	18.28(16.58,20.34)	18.12^***^	−0.04^*^
		> 70	43.23(39.70,46.80)	50.97(48.08,53.60)	48.76(45.87,50.99)	42.76(39.62,44.60)	42.82(39.38,44.89)	43.19(38.68,46.98)	35.62^***^	−0.23^*^
Global	Mortality									
		0–14	0.28(0.23,0.35)	0.28(0.24,0.32)	0.20(0.18,0.24)	0.18(0.16,0.21)	0.18(0.15,0.20)	0.17(0.14,0.20)	0.20^*^	−0.002^*^
		15–49	2.89(2.50,3.34)	3.44(3.16,3.74)	2.28(2.12,2.47)	1.60(1.51,1.73)	1.56(1.44,1.71)	1.54(1.35,1.74)	1.12^*^	0.004^***^
		50–69	26.85(23.91,30.46)	29.41(27.36,31.56)	21.81(20.48,23.23)	16.18(15.49,16.88)	15.49(14.64,16.38)	15.77(14.33,17.25)	17.37^***^	−0.05^***^
		> 70	48.98(44.98,52.91)	56.81(53.24,60.06)	50.43(47.01,52.83)	44.74(41.41,46.73)	43.64(39.92,45.93)	43.84(39.65,47.17)	41.62^***^	−0.18^***^
	1-MIR									
		0–14	0.00(0.00,0.00)	0.00(0.00,0.02)	0.07(0.04,0.12)	0.07(0.02,0.15)	0.08(0.05,0.17)	0.12(0.10,0.18)	0.17^*^	0.003^*^
		15–49	0.11(0.11,0.11)	0.11(0.12,0.10)	0.20(0.22,0.18)	0.17(0.18,0.17)	0.21(0.23,0.20)	0.23(0.22,0.23)	0.14^*^	0.002^***^
		50–69	0.05(0.05,0.05)	0.06(0.07,0.06)	0.13(0.15,0.12)	0.11(0.12,0.11)	0.14(0.15,0.13)	0.14(0.14,0.15)	0.03^*^	0.001^***^
		> 70	0.00(0.00,0.00)	0.00(0.00,0.00)	0.00(0.00,0.00)	0.00(0.00,0.00)	0.00(0.00,0.00)	0.00(0.00,0.00)	–	–

Data in parentheses are 95% uncertainty intervals

*CEEECA* Central Europe, Eastern Europe, and Central Asia, *HI* High Income, *LAC* Latin America and Caribbean, *NAME* North Africa and Middle East, *SA* South Asia, *SAEAO* Southeast Asia, East Asia, and Oceania, *SSA* Sub-Saharan Africa, *MIR *Mortality-to-incidence Ratio, *LGCM *Latent growth curve model

^*^ *p*<0.05, ^**^ *p*>0.05, ^***^ *p*<0.001

^a^Not fitted LGCM, due to inadequate number of countries

**Fig. 4:**
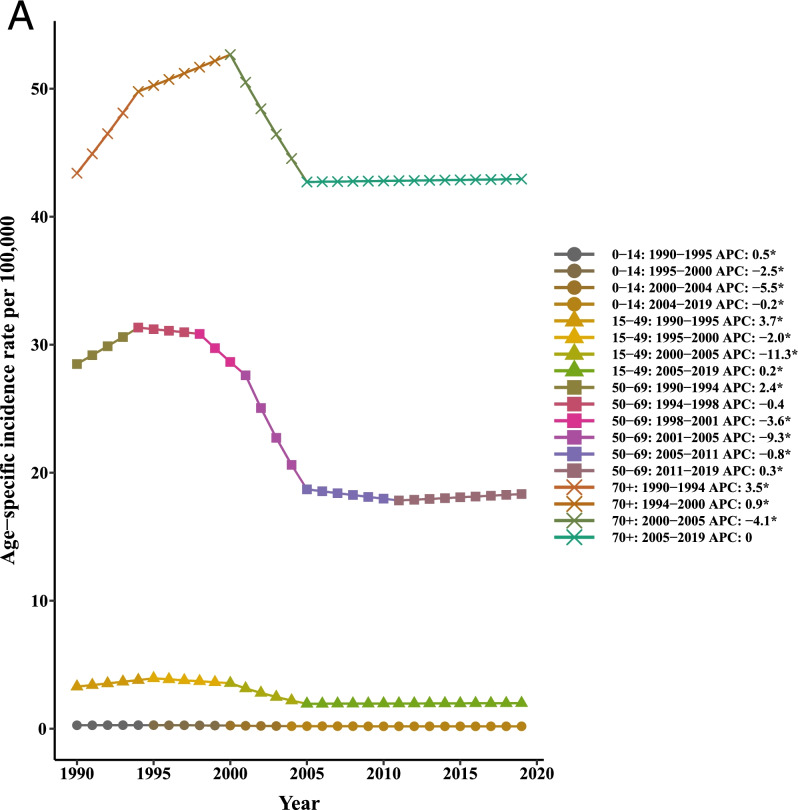

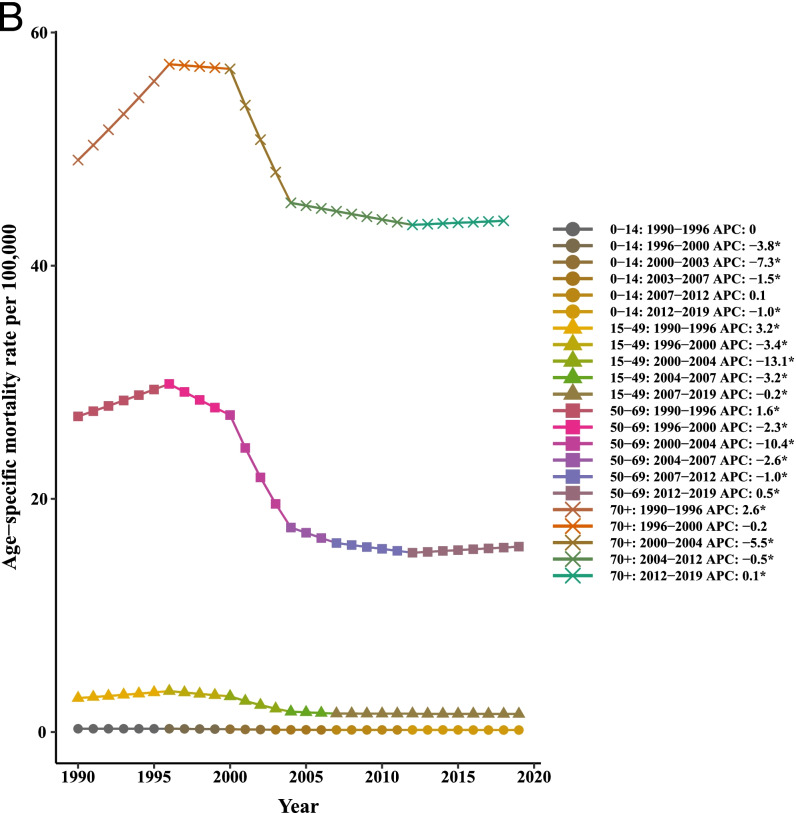

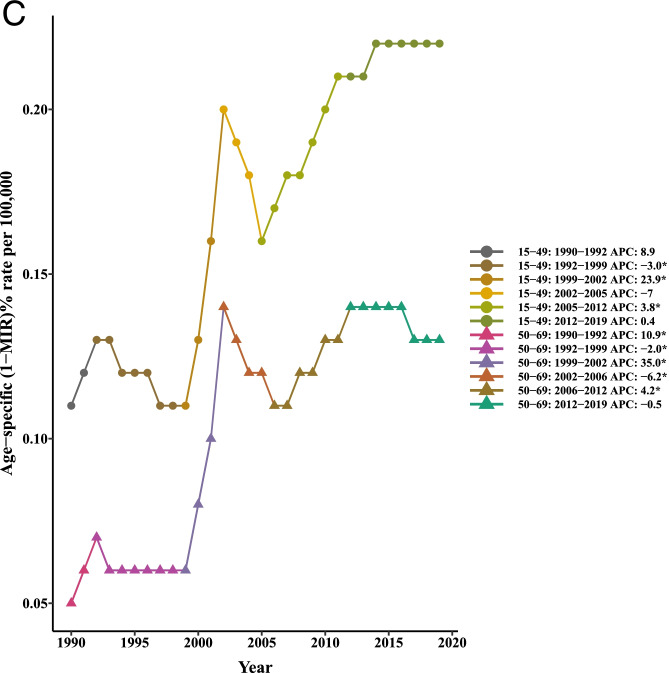
Joinpoint regression analyses of trends in mean trends of liver cancer (**A**) incidence, (**B**) mortality, and (**C**) [1-MIR] per 100,000 by age group worldwide, 1990–2019

Table 4 and Fig. 4 summarized the results of the joinpoint regression analyses of the age-specific burden of LC. The AAPCs of mortality were significantly negative for all age groups of 0–14 (− 1.80, 95% CI: − 2.00, − 1.60), 15–49 (− 2.10, 95% CI: − 2.50, − 1.80), 50–69 (− 1.80, 95% CI: − 2.10, − 1.60), and 70+ (− 0.40, 95% CI:-0.50, − 0.30) in the period 1990–2019. Similar findings were observed for trends of liver cancer incidence in persons aged 0–14 (− 1.20, 95% CI: − 1.40, − 1.10), 15–49 (− 1.70, 95% CI: − 1.90, − 1.50), and 50–69 (− 1.50, 95% CI: − 1.60, − 1.40). However, the AAPC values of 2.40 (95% CI: 0.30, 4.50) and 3.20 (95% CI: 1.70, 4.80) was seen for age groups 15–49 and 50–69, respectively. It should be noted that the APCs were not calculated in the age groups 0–14 and + 70 since most of the data for [I-MIR] were zero.

**Table 4:** Joinpoint trend analysis of liver cancer incidence, mortality, and 1-MIR rates worldwide by age group, 1990–2019

Measure	Trend	0–14		15–49		50–69		70+	
		Year	APC (95%CI)	Year	APC (95%CI)	Year	APC (95%CI)	Year	APC (95%CI)
Mortality									
	Trend1	1990–1996	0(−0.30, 0.20)	1990–1996	3.20*(2.70, 3.70)	1990–1996	1.60*(1.30, 2.00)	1990–1996	2.60*(2.40, 2.80)
	Trend2	1996–2000	−3.80*(−4.50, −3.20)	1996–2000	−3.40*(−4.70, −20)	1996–2000	−2.30*(−3.20, −1.40)	1996–2000	−0.20(− 0.70, 0.30)
	Trend3	2000–2003	−7.30*(−8.60, −6.00)	2000–2004	− 13.10*(− 14.30, −11.90)	2000–2004	− 10.40*(− 11.20, −9.60)	2000–2004	−5.50*(−6.00, −5.00)
	Trend4	2003–2007	−1.50*(−2.20, − 0.80)	2004–2007	− 3.20*(−5.80, − 0.50)	2004–2007	−2.60*(− 4.40, − 0.80)	2004–2019	−0.50*(− 0.70, − 0.40)
	Trend5	2007–2012	0.10(− 0.40, 0.50)	2007–2019	−0.20*(− 0.30, 00)	2007–2012	− 1.0*(− 1.60, − 0.50)		
	Trend6	2012–2019	− 1.00*(− 1.20, − 0.80)			2012–2019	0.50*(0.20, 0.70)		
	AAPC	1990–2019	− 1.80*(− 2.00, − 1.60)	1990–2019	− 2.10*(− 2.50, − 1.80)	1990–2019	− 1.80*(− 2.10, − 1.60)	1990–2019	− 0.40*(− 0.50, − 0.30)
Incidence									
	Trend1	1990–1995	0.50*(0.10, 1.00)	1990–1995	3.70*(3.20, 4.10)	1990–1994	2.4*(2.10, 2.7)	1990–1994	3.50*(3.20, 3.80)
	Trend2	1995–2000	−2.50*(− 3.10, − 1.90)	1995–2000	−2.00*(− 2.60, − 1.40)	1994–1998	− 0.40(− 0.80, 0.00)	1994–2000	0.90*(0.700, 1.10)
	Trend3	2000–2004	− 5.50*(− 6.40, − 4.60)	2000–2005	− 11.30*(− 11.90, − 10.80)	1998–2001	− 3.60*(− 4.50, − 2.80)	2000–2005	−4.10*(− 4.40, − 3.80)
	Trend4	2004–2019	− 0.20*(− 0.30, − 0.10)	2005–2019	0.20*(0.10, 0.30)	2001–2005	−9.30*(− 9.70, − 8.90)	2005–2019	0.00(0.00, 0.10)
	Trend5					2005–2011	− 0.80*(− 1.00, − 0.60)		
	Trend6					2011–2019	0.30*(0.30, 0.40)		
	AAPC	1990–2019	− 1.20*(− 1.40, − 1.10)	1990–2019	−1.70*(− 1.90, − 1.50)	1990–2019	−1.50*(− 1.60, − 1.40)	1990–2019	0.00(− 0.10, 0.00)
1-MIR									
	Trend1			1990–1992	8.90(−4.50, 24.20)	1990–1992	10.90*(0.100, 22.90)		
	Trend2			1992–1999	− 3.00*(− 5.10, − 0.80)	1992–1999	− 2.00*(− 3.70, − 0.30)		
	Trend3			1999–2002	23.90*(8.70, 41.20)	1999–2002	35.00*(21.80, 49.50)		
	Trend4			2002–2005	−7.00(−18.40, 6.00)	2002–2006	−6.20*(− 10.90, − 1.30)		
	Trend5			2005–2012	3.80*(1.60, 6.20)	2006–2012	4.20*(1.80, 6.60)		
	Trend6			2012–2019	0.40(−1.30, 2.20)	2012–2019	−0.50(− 1.90, 0.90)		
	AAPC			1990–2019	2.40*(0.30, 4.50)	1990–2019	3.20*(1.70, 4.80)		

*APC* Annual Percentage Change, *AAPC* Average Annual Percent Change, *CI *Confidence Interval, *MIR *Mortality-to-incidence Ratio

*Significantly different from 0 at alpha = 0.05 (*p* < 0.05). There are 1+(number of trend) joinpoints for each model

In the next step of analysis, we employed 4-level modeling approach to understand how the development status (i.e. HDI) affect the incidence, mortality, and [1-MIR] over time by accounting for the clustering nature of the GBD data and the response of the outcome variables. We first proceeded with explaining variability in the random parameters across countries, regions, and super-regions to address the research question: are there any differences in development among groups of countries or regions or super-regions. According to our results, substantial significant variability within the level 2 units (i.e. countries) in terms of ASIR and ASMR, suggested remarkable heterogeneity between countries from the same super-region in 1990 (*p* < 0.05). In other words, the variations in mean incidence and mortality across countries can be explained by the differences in the socioeconomic status. Nonetheless, the variances of random components were not statistically significant in region and super-region levels (*p* > 0.05). Likewise, looking at the estimated ICCs in the empty multilevel models (without predictor variable), we found that the variations between countries were responsible for around 88.2 and 88.07% of the overall variability respectively in repeated measures of ASIR and ASMR (Table 5). This might be because of the difference at cluster level or unmeasured country factors/characteristics. In particular, the fairly large ICC values implied plausibility of similarity among repeated measures of observations in a cluster. On the other hand, although the least ICCs was observed at either region or super-region levels for ASIR and ASMR, accounting for their unexplained variability in the models, one can use more information in estimating parameters. Overall, the existence of great ICC value in the empty model revealed that we did better in using longitudinal multilevel modeling as a proper approach to estimate model parameters rather than the standard single-level mixed effects regression model. Of important, due to very small values of ICC related to countries, regions, and super-regions (around 0) in MIR, there was trivial degree of correlation among observations within each cluster, justifying they would not affect one another nor would they be similar (i.e., no homogeneity). This signified that the multilevel model was not applicable for this metric. By adding the development status to the empty models, the estimated significant positive slopes of time demonstrated that the trends in ASIR and ASMR increased relatively stable in the period 1990 to 2019 (*p* < 0.05). Also, there are statistically noticeable difference between less-developed and more developed countries in terms of means ASIR and ASMR (p < 0.05). Indeed, the estimates of − 1.36 and − 1.07 for development status suggest that the more developed countries had, on average, 1.36 incidence and 1.07 mortality per 100,000 people which were lower compared to the less-developed counterparts. As such, no statistically significant interaction reported for development status and time, declares that the growth trajectory of both means of ASIR and ASMR for more developed and less-developed nations were parallel over time (*p* > 0.05). Clearly, this means the effect of development status remained almost constant during 1990–2019.

**Table 5:** Longitudinal multilevel analysis testing the impact of development status on the incidence and mortality from liver cancer, 1990-2019

Metric	Parameter		Estimate	SE	*P* value
Incidence	Intercept		5.67	0.78	0.007^*^
	Time		0.02	0.004	0.025^*^
	Development status				
		More Developed	-1.36	0.22	0.012^*^
		Less-Developed	Reference	-	-
	Development status × time		0.02	0.01	0.157^**^
	Random effects				
		$\sigma_{u_{0i}}^2$ (between countries)	63.28	6.84	0.002^*^
		$\sigma_{\upsilon0}^2$ (between regions)	3.96	3.44	0.283^**^
		$\sigma_{\omega0}^2$ (between super-regions)	0.10	0.08	0.262^**^
	**ICC (%)**				
	Country level	88.2	-	-	-
	Region level	5.5	-	-	-
	Super-region level	0.13	-	-	-
Mortality	Intercept		5.80	0.81	0.007^*^
	Time		0.02	0.005	0.043^*^
	Development status				
		More Developed	-1.07	0.25	0.038^*^
		Less-Developed	Reference		
	Development status × time		0.03	0.01	0.083^**^
	Random effects				
		$\sigma_{u_{0i}}^2$ (between countries)	69.93	7.57	0.002^*^
		$\sigma_{\upsilon0}^2$ (between regions)	4.01	3.66	0.296^**^
		$\sigma_{\omega0}^2$ (between super-regions)	0.11	0.10	0.294^**^
	**ICC (%)**				
	Country level	88.07	-	-	-
	Region level	5.06	-	-	-
	Super-region level	0.14	-	-	-

*ICC* Intraclass Correlation Coefficient; *SE* Standard Error, ^*^p < 0.05, ^**^p > 0.05

## Discussion

Over the past decades, liver cancer has been known as one of the primary lethal neoplasms which has a widespread distribution across the globe with a serious threat to the public health [33]. The present paper provided strong evidence that the patterns of LC [1-MIR] rates and its components were extremely diverse among world super-regions for both genders from 1990 through 2019. One reason that may explain this disparity would be differences in distributions of risk factors in various populations. In particular, SAEAO was the only super-region with the largest ASIRs and ASMRs related to LC versus other regions over the last 30 years. Similar findings were noted in Lin et al. announced that East Asia and Southeast Asia had the highest liver cancer burden among geographic regions, accounting for about 85% of the global incidents and deaths from 1990 to 2017 [34]. These results highlight that LC in these countries was likely to be remained as a major public health problem, albeit with a change in the distribution of cases around the world.

With respect to the time period, a generally declining pattern was observed for ASIR and ASMR in SAEAO and LAC with the steepest slope, which benefited from primary and secondary prevention, new treatments development, and improvement of antiviral therapy. In line with what we observed for the trends in these regions, a comparative analysis of temporal trends in LC incidence rates among Eastern and Southeastern Asian countries reported that the rates decreased between 1983 and 2007 [35]. Also, the study of Jemal et al. found that since the early 1960s to 2008, the incidence rates in most of the Asia populations (including China and Korea) fell which is thought to reflect reduction in transmission of hepatitis B virus (HBV). Conversely, they approved that LC incidence and mortality rates increased in Oceania due to widespread hepatitis C virus (HCV) infection [36]. The ongoing decrements in mortality of LC in these continents can be partially attributed to the expansion in access to healthcare services namely primary care, health promotion actions, and improvements in socioeconomic aspects.

Another striking finding was that the largest increasing time trends in ASIR and ASMR was observed in HI and SSA till 2000, followed by a monotone slight decline during 2000–2019. This result reminds us that liver cancer should not be placed at a lower priority in disease prevention and treatment in those regions. Although the reasons behind the growing patterns in the LC incidence and mortality in these countries are absolutely unclear, a part of the trend, especially in the initial years of the study, may be attributed to economic development, increased exposure to environmental risk factors, lifestyle habits, and a rise in the number of patients registered. Similar findings have been reported in other recently published paper showing that the incidence and mortality have increased substantially from 1982 to 2014/2015 [37]. Population aging and growth, high alcohol consumption, and low intake of vegetables and fruit likely play a small, or minimal role in upward trend of LC among these populations.

Looking at the temporal patters of ASIR and ASMR in SA, changes were not as pronounced as those observed in other regions over the entire reporting period. Furthermore, there was a gentle decline of ASIR and ASMR in these populations from 2002 to 2019. LGCMs also showed that the initial values of ASIR and ASMR at the start of this study differed across the continents, probably because of unequal healthcare coverage and variations in compliance to regular screening and necessary treatments. Moreover, countries of CEEECA had the highest average rates of change in incidence and mortality compared to other regions, though this may be due—at least in part- a different proportion and variable time trends of LC. Such findings can be justified by a report in Europe where there was between country variability in mortality patterns and trends since 1970s continued over the 1990s but levelled off during the 2000s [38].

In terms of gender, concordant with those published by previous epidemiological studies [33, 34], the ASIR and ASMR attributed to LC in males were higher than that of females across different super-regions. However, the reasons for this gender time-dependent changes are complex and may stem from differences in genetic risk factors, hormonal changes, metabolic factors, treatments received, compliance with antiviral therapies, and tumor biology [39]. Based on the LGCMs, it was seen that the overall mean levels of initial ASIR and ASMR in males were substantially greater in comparison to females of all super-regions in 1990. Nevertheless, the slopes of ASIRs and ASMRs in these regions illustrated that the growth trajectories for both males and females were almost similar over the period 1990–2019 which is broadly in accordance with some reports [40].

Among both sexes, the average rates of change of ASIR and ASMR were highest and lowest in CEEECA and NAME, respectively over time. Although to our knowledge, there were no published articles covering the period of our study, our findings regarding gender differences in LC incidence and mortality rates are confirmed by a number of available literature. On the basis of GLOBOCAN 2018 estimates, some papers suggested that Eastern Asia, South Eastern Asia and Northern and Western Africa suffered from the highest incidence among both sexes and highest mortality in females. Likewise, the highest mortality was found in Eastern Asia and Western Africa in males. Accordingly, the lowest incidence and mortality tended to predominate in South Central Asia, Western Asia, Central Europe, and Eastern Europe for either gender [1, 41]. Based on a report in China, the unfavorable trends of LC in men and women was more likely to be exposed to risk factors such as tobacco use, alcohol abuse, and income inequality that interactions between them may have led to an increment in risk of LC [42]. In a recent incidence and mortality descriptive analysis from GLOBOCAN 2018, the author observed that few regions, like East Asia and South East Asia had higher disparities in men and women, whilst Central America and South America had lesser male-female disparities in terms of ASIR and ASMR as well as counts [43]. Another research performed a comprehensive analysis based on the GBD study 2017 assessed the global temporal trends in liver cancer incidence and mortality during 1990–2017. They observed that East Asia had the highest ASIR and ASMR for both genders and the latter was as high as 40.4/100,000 among males. The biggest difference in the ASMR of men and women was revealed in High-income Asia Pacific. Contrary to our results, in their research, the incidence and mortality have grown in males but reduced in females, suggesting that this gap might broaden in the future. Further, they found that the incidence and mortality rates were greater in females compared to males in the Latin American countries over the last 28 years. Nonetheless, more researches are required to confirm these conjectures [34]. In a recent study by Li et al., the authors provided evidence that hepatocellular carcinoma (HCC) predominantly may affect males with incidence twofold to fourfold more common in males than females [44]. Petrick et al. using the joinpoint analysis demonstrated the incidence rates of LC in males were two to three times greater than in females among countries of Africa and the Americans. Compatibly, European countries had the highest variability in incidence between sexes. In their opinion, the higher rates in men is most likely due to higher prevalence of known LC risk factors such as alcohol consumption, smoking, differences in sex steroid hormones, immune responses and/or epigenetic differences between females and males [40]. Nevertheless, we wondered that whether the underlying causes might contribute to this gender disparity. Hence, further examinations are warranted to falsify such these hypotheses. Furthermore, with respect to time trends, it is apparent that there were considerable gaps between males and females in ASIR and ASMR within super-regions; notably, the gaps remained constant until the ending year of this study. Although we could find no regional studies that examined the gap between genders for temporal trends of liver cancer burden, it is probable that differences in some factors such as education, income, and employment are conducive to the gender gaps. Nonetheless, future studies require to find further factors related to the differences and explore ways to narrow the gender gap over time. Globally, it was observed about 27.5 and 33.3% reductions in incidence and mortality rates were occurred in total populations, respectively during a 30-year follow-up. This might be due in part to preventive measures including self-examination, public education, and development of new multiple treatment options and better control in past decades.

Particularly, the rates at the global level showed that the LC incidence and mortality rates among males declined by almost 25.7 and 32.3%, respectively from 1990 to 2019. Whilst, the drop among females in incidence and mortality rates were 30.4 and 35.1%, respectively in this 30-year period. Clearly, these results imply that although the males had higher rates, the females have experienced a further reduction in both incidence and mortality throughout the observation period which is encouraging. The reason of the more decrement was not completely investigated. Nonetheless, various explanations could be proposed for this phenomenon. First, steeper overall improvement in LC in women than men is potentially associated with the success of public health effects and growing the awareness of LC risk. Second, females were significantly more possibly to undergo liver transplant. Ultimately, females with LC were also more likely to undergo screening than males with the disease.

Based upon the data from the GBD study 2019, the patterns for age-specific trends differed across regions. It was illustrated that much more similar rates of change in LC incidence and mortality in the respective continents occurred among persons aged 0–14 and 15–49 so that the growth trajectories for these age groups remained fixed in all regions over the whole study time 1990–2019. In accordance with a previously reported study, we have seen that the LC incidence and mortality rates in the group aged 50–69 and = > 70 years were higher than that of the 15–49 and 0–14 age groups throughout the study period [42].

The results obtained from LGC analysis demonstrated that the highest initial values of incidence and mortality by the year 1990 were belonged to SAEAO among individuals aged 70 years or older and the lowest were recorded in NAME and HI in individuals aged below 14 years, respectively, demonstrating older people experienced a heavier disease burden due to LC. Subsequently, individuals aged 70+ years had the most average rates of change in incidence and mortality among CEEECA countries, while the lowest were occurred in SSA for the same age group. The evidence indicates that LC has a strong relationship with age such that the aging could contribute to the increasing disease burden of LC. Furthermore, both age-specific incidence and mortality trends showed the most rapid increases among HI (from 1990 to 2006) and CEEECA (from 1999 to 2010) countries compared to the others in those aged 70 and over; therefore calling for better preventive measures in these countries. Regarding the patterns of incidence and mortality for patients aged 50–69 years, with exception of SAEAO, the trends were relatively steady during the interval 1990–2019. In parallel, Zhai et al. using GBD data 2017 observed that the incidence of primary liver cancer was higher in middle-aged people among high and middle income countries [45]. As well, Lee et al. in Korea as a high-income country concluded that age-specific incidence of LC incremented gradually with age and those aged over 80 years possess the highest incidence, and follows a similar trends in some Asia countries including China, Japan. Philippines, Singapore, and Thailand [46]. In another study, researchers found that trends of mortality from LC in the United States and Australia were similar to those observed in Central and Northern European countries, indicating upward trends in individuals aged 45–64 years [37]. A study by Liu and colleagues assessed temporal patterns in LC incidence and death by sex, age, and etiology in China from 1990 to 2017. They reported the most pronounced increase in ASIR and ASMR were among older people aged ≥ 70 years, whereas it remained stable in the other two age groups (15–49 and 50–69 years) during 2004–2014, which is consistent with our results. The rises were partly due to the increase in healthcare availability in recent years [47]. On the contrary, a Chinese study with a follow-up period of 30 years documented that people between ages of 15 and 49 years had a greater reduction in incidence and deaths from 1990 to 2019 compared with people at 50–69 years of age [27]. Overall, temporal trends in LC incidence and mortality rates among all age groups have been decreasing in the world throughout the study period which is reflective of improved therapeutic strategies. Interestingly, the 15–49 years old group had the highest percentage decrease in incidence and mortality accounted for about 38.7 and 46.7% respectively compared to other age groups. According to some publications, age is a firm prognosis in many malignant cancers; nevertheless, the association between aging and cancer is still not clear [47, 48]. It was confirmed that genome alternations including DNA mutations accumulate with age. A recent study delineate the liver greatly affected by aging in terms of its function and structure and contains regulation of the tumorigenicity and development of HCC through liver microenvironment modulation which might facilitate tumor formation and progression [49].

In the current article, [1-MIR] has been utilized as an approximation of the five-year relative survival clarified by a number of evidence-based studies [3, 4, 50] that can be reliably applied. In general, the geographic continent analysis provided that the super-regions were different in time patterns of [1-MIR] representing regional varieties in health care systems, screening cancer, effective treatment, and prognosis for LC. Of the 7 super-regions compared, we discovered that HI had the highest [1-MIR] rates for both genders, males, and the 0–14 years old population from 1990 to 2019. The values of [1-MIRs] in both genders and males suggest the five-year relative survival rates were moderately poor in these countries which might be due to high recurrence rates and a more advanced tumor stage at diagnosis. In contrast, subjects of 0–14 years have experienced almost superior five-year relative survival rates in comparison to other three age categories such that the rate was about 61% at the end of the follow-up years. A possible explanation includes that, children 0–14 years of age had the most favorable prognosis; plus, poorer biological behavior among younger people might be compensated by better liver function, more aggressive therapy, and faster recovery contributing to longer survival [51]. An alternative elucidation could be that, tumors from age groups with favorable survival are biologically less aggressive compared with those tumors from age groups with lower survival. Our findings are in agreement with some previous reports in the United States [51] and Taiwan [52]. There is yet a descriptive study to be undertaken using GLOBOCAN 2012 database that implicated high rates of MIRs in less developed regions regardless of age and gender. That study also reported North America had the lowest MIR (0.82), while Latin America and Caribbean had the highest MIRs (1.04). These different results may thus contribute to the treatment familiarity, surgical, and invasive procedure techniques [53]. Globally, the overall picture of the observed findings of [1-MIR] rates indicated the rates were not considerable and almost all of them were around 0 for either sex or different age categories, implying a poor five-year relative survival rate and worsening cancer care. Constant development of systemic therapies especially in patients with an advanced stage, improving the early detection of LC, enhancing surgical techniques, postoperative care, and timely surgical treatment are key factors in improving the survival rate for patients with LC. However, since the [1-MIR] is an indirect measure and also an overestimation of LC five-year relative survival with at least 10% in some countries, the results should be interpreted with caution [54]. Notably, due to the limited information in this context, further follow-up studies of the [1-MIR] from liver cancer would be warranted in various parts of the world.

Concomitantly, joinpoint regression analysis demonstrated that liver cancer incidence and mortality have declined significantly in both males and females over the past two decades. Likewise, similar trends in incidence and mortality were observed across age groups. [1-MIR], however, exhibited significant upward trends in age and sex groups during the study period. This means that the mortality rate was declining over time more quickly compared to the incidence rate. Few studies have examined the trend of liver cancer in the world as a whole in terms of incidence, mortality and [1-MIR]. A study by Lin et al. on GBD 2017 data showed that the mortality rate and incidence rate increased between 1990 and 2017 [34]. That is completely in contrast to our study. One reason could be that the trend incidence and mortality assessments in their study were only based on the APC and not the AAPC. Therefore, the overall trend of mortality and incidence rate in their study was incorrectly reported. To examine the incidence and mortality of a cancer over the years, it is necessary to report AAPCs to determine whether the trend has changed significantly. APC may decrease in one period and increase in another, but AAPC may not show any significant change. In another paper conducted by Huang et al., an upward in incidence rates was revealed in many countries between 1980 and 2017. However, a global analysis of trends in incidence was not conducted in this study [33]. In general, when examining the trends of incidence and mortality worldwide, the correct statistical methodology should be considered. Whereas in the above studies, AAPC was reported for different regions but not specifically for global. One of the advantages of the AAPC criteria is that it can provide information about how incidence and mortality have changed over time. This may help explain global differences in the liver cancer trends.

The ICC estimates in unconditional multilevel models (without any independent variable) declared a high degree of clustering in the GBD data so that the majority of the variation lies at the country level. Indeed, 88% of total variations in repeated measures of incidence and mortality are due to differences between countries. The differences might be attributed to some factors such as income inequality, access to the health care facilities, and clinical and behavioral characteristics. The current study employed 4-level random intercept multilevel hierarchical models to explore whether there are significant differences between more developed and less-developed countries in terms of mean incidence and mortality during the specified time interval. By using these models, the data hierarchical structure can be specified by varying the random effects at each level of the hierarchy. In these hierarchies, there is vital information to identify better and targeted policy formulation. The results obtained from fitting multilevel model exhibited that the development status had a statistically significant negative effect on both LC incidence and mortality rates such that the means of these metrics in more developed countries were significantly lower than those in less-developed ones. This observation demonstrates the importance of socio-economic factors in declining trends of LC burden over the long-term. In 2012, it was reported approximately 95% of total incidence and 96% of all deaths from LC occurred in less developed areas. These countries are often in the process of industrialization which can affect all aspects of lives like health [41]. On the other hand, HBV causes 2/3 cases of LC in less-developed countries, while only 1/4 cases in more developed ones. Moreover, HCV in less-developed countries (such as Egypt and Liberia) is a fairly less important cause of LC so that it causes 1/8 cases, whilst almost 1/2 cases in more developed countries. Thus, HBV and HCV can be strong risk factors for LC in less-developed countries [55]. Our findings are in keeping with recently published cross-sectional studies that found a negative correlation between LC incidence and mortality and HDI levels. The authors confirmed that the countries with higher levels of HDI were related with lower LC incidence and mortality rates [12, 56–58]. The correlation is more likely caused by promotion of aggressive clinical guidelines, the development of sophisticated technology, the improvement of socioeconomic status, and increase in average academic years. On the other hand, health care disparities and availability of essential technology and resources for prevention, may result in the substantial gap between the more developed and less-developed populations in terms of incidence and mortality. This is a worrying message for health policy makers. Consequently, to close the gap, both wealthier and poorer parts of the world should be focused on promoting heightened awareness, preventive measures, earlier detection, better patient care, screening, and effective treatment.

The strengths of the present study are worthy of mention. First, this work benefitted from its longitudinal design, with a notable number of repeated measurements, and a lengthy follow-up which can provide more evidence of a causal association. The novelty of this study beyond previous researches on the relationship of HDI and burden of LC is its use of multilevel modelling approach to attribute the variability of random effects across various levels of hierarchical structure. Of note, the consideration of random effects at each level makes it possible reliable estimates by reflecting hierarchy, thereby correcting the underestimation of standard errors. However, such variations cannot be captured in traditional repeated measures analysis such as analysis of variance (ANOVA). Furthermore, since liver cancer tumors have high lethality, the [1-MIR] appears to be as an alternative fairly accurate simple measure for five-year relative survival rate. Despite these strengths, a number of limitations of this research should be recognized. First of all, this report was based on the most recently updated data from the GBD 2019 in which ascertainment bias in cancer registries may lead to a problem in data accuracy. Second, although we tried to consider nearly 204 countries and territories, missing data of liver cancer incidence and mortality reduced our cases to approximately 184 countries due to limited information sources. Finally, owing to the lack of longitudinal information of important risk factors such as HBV and HCV infections, occupational exposures, chemical, and pollution exposures, alcohol drinking, and tobacco use, the effects of them on liver cancer outcomes were not examined. These factors may play important roles in explaining and detecting trend of LC incidence and mortality rates. Consequently, we suggest further future longitudinal studies to determine the impact of the underlying factors on the liver cancer outcomes in different regions and countries using random slope coefficient multilevel model.

## Conclusions

Collectively, the body of the current evidence exhibited the steady but slow declining patterns of LC incidence and mortality at the global level over the past 30 years. Meanwhile, the observed disparity of LC incidence and mortality time trends between countries can be attributed to unequal medical levels and resources. Accordingly, although in the current analysis an increasing trend in [1-MIR] was seen during 1990–2019, the small values suggest that the five-year relative survival rate was poor. Its reason might be less proven screening modalities available for early screening and identification of LC. Besides, this was the first study to assess longitudinal relationship between HDI and burden of LC taking into account the multilevel GBD data structure. When the observations are correlated in the cluster, regardless of clustering may result in biasedly estimated variances. Whilst, applying multilevel model, the bias in the estimates can be corrected. Through this model, we found that the incidence rates of LC, as well as mortality rates, have declined with a slight slope in more developed nations. Rapid progress in economic development, optimization of public health policy, improvements in treatment, increase medical insurance, and management of patients with LC may account for a substantial proportion of the favorable trends.

## Data Availability

The datasets analyzed during the current study are publicly available in http://ghdx.healthdata.org/gbd-results-tool and http://hdr.undp.org/en.

## Abbreviations

LC: Liver cancer
MIR: Mortality-to-incidence ratio
HDI: Human development index
GNI: Gross national income
GBD: Global burden of disease
GHDx: Global health data exchange
IHME: Institute for health metrics and evaluation
SDI: Socio-demographic index
UI: uncertainty interval
LGCM: Latent growth curve model
APC: Annual percent change
AAPC: Average annual percent change
CI: Confidence interval
GLMM: Generalized linear mixed models
ICC: Intraclass correlation coefficient
REML: Restricted maximum likelihood estimation
ASIR: Age-standardized incidence rate
ASMR: Age-standardized mortality rate
SAEAO: Southeast Asia, East Asia, and Oceania
SA: South Asia
LAC: Latin America and Caribbean
SSA: Sub-Saharan Africa
HI: High Income
CEEECA: Central Europe, Eastern Europe, and Central Asia
NAME: North Africa and Middle East
HBV: hepatitis B virus
HCV: hepatitis C virus
